# Unidirectional molecular rotary motor with remotely switchable rotation direction

**DOI:** 10.1126/sciadv.adt8008

**Published:** 2025-05-16

**Authors:** Kamil Szychta, Wojciech Danowski, Joanna Jankowska

**Affiliations:** ^1^ Faculty of Physics, University of Warsaw, Warsaw 02-093, Poland.; ^2^ Faculty of Chemistry, University of Warsaw, Warsaw 02-093, Poland.

## Abstract

Light-driven rotary motors allow direct transformation of light energy into rotary motion at the nanoscale, giving rise to countless emerging applications in molecular engineering. The key feature enabling the unidirectional rotation and controlling its direction is the motor chirality, a factor hard to modify postsynthetically. Here, we propose a motor architecture, E-motor, whose operation direction can be switched remotely with an electric field pulse, without the need for chemical intervention. Our study relies on quantum chemical calculations and nonadiabatic molecular dynamics simulations performed for a specifically tailored system, PFCN, designed to provide illustration for the proposed motor type. We show that the PFCN chirality depends on the orientation of a covalently bound polar switching unit, which can be controlled with the electric field. At the same time, the proposed system manifests all characteristic photophysical properties of a molecular motor, and its set chirality is preserved during operation in the absence of the field.

## Teaser

A redesigned molecular motor can be switched with an electric field to change the rotation direction.

## INTRODUCTION

Molecular rotary motors belong to a growing family of functional molecular devices capable of transforming chemical, electric, or light energy into mechanical work (*1*–*4*), offering unprecedented means for controlling motion at the nanoscale and paving the way for design of interactive, “smart” molecular systems and materials for countless applications (*5*–*17*). Among other molecular systems of such kind (*3*, *7*, *18*–*20*), light-driven rotary motors characterize with continuous unidirectional rotary motion under a flux of photons of energy matching the electronic absorption spectrum of the motor. In the course of this motion, a part of the molecular system considered static, the “stator,” constitutes a reference framework for the other, mobile part, the “rotor,” and the statistically dominant unidirectionality of their mutual rotation stems from the inherent system chirality. The historically first and the nowadays most populated group of molecular motors are overcrowded alkenes, in which the light-triggered rotation occurs about a double C═C bond. It is important to note that, amid the overcrowded alkenes, three generations of motors can be distinguished (*21*): (i) the first generation, in which stationary and rotating parts of the molecule are identical, both having same-oriented stereogenic centers; (ii) the second generation, in which two halves of the motor are different and both, or only one of them, have a stereogenic center; and (iii) the third generation, in which, contrary to the previous two, the motor consists of one pseudochiral stator and two rotors, bonded on its opposite sites. From the perspective of this work, the axial chirality of molecular rotary motors, i.e., their preference for adopting one of the two possible relative orientations of the rotating and stationary parts, plays an essential role. The motor chirality enables the unidirectional rotation and controls its direction (*18*). The origin of this effect lies, on the one hand, in the presence of a point stereogenic center (chiral or pseudochiral) and, on the other, in the sterically crowded surrounding of the central double bond about which the rotation occurs. Because of the fundamentally chemical nature of both factors, artificial motors’ chirality and resulting rotation direction can be controlled almost exclusively at the synthesis stage, usually through separation of enantiomers from a racemic mixture of the synthesized product (*21*) or by taking advantage of the asymmetric synthesis routes (*22*, *23*). At the same time, over the years, several attempts to reverse the rotation direction of a once prepared optically driven motor have been made. First, the circularly polarized light was used to control the motion of an inherently nonchiral molecular rotor (*24*); however, the overall prevalence of one direction of rotation over the other did not exceed 1%. Alternatively, postsynthetic base-catalyzed epimerization (*25*) and achiral-host–chiral-guest noncovalent interactions (*26*) were used to switch between clockwise (CW)/counterclockwise (CCW) motor operation. A similar, catalytic strategy has been also recently used to control rotation about a single bond in a chemically powered molecular rotor (*27*). The latter approaches, however, all involve a full-scale chemical intervention. Eventually, a robust, real-time control of rotation in molecular motors still remains a major challenge (*28*).

**Fig. 1.**
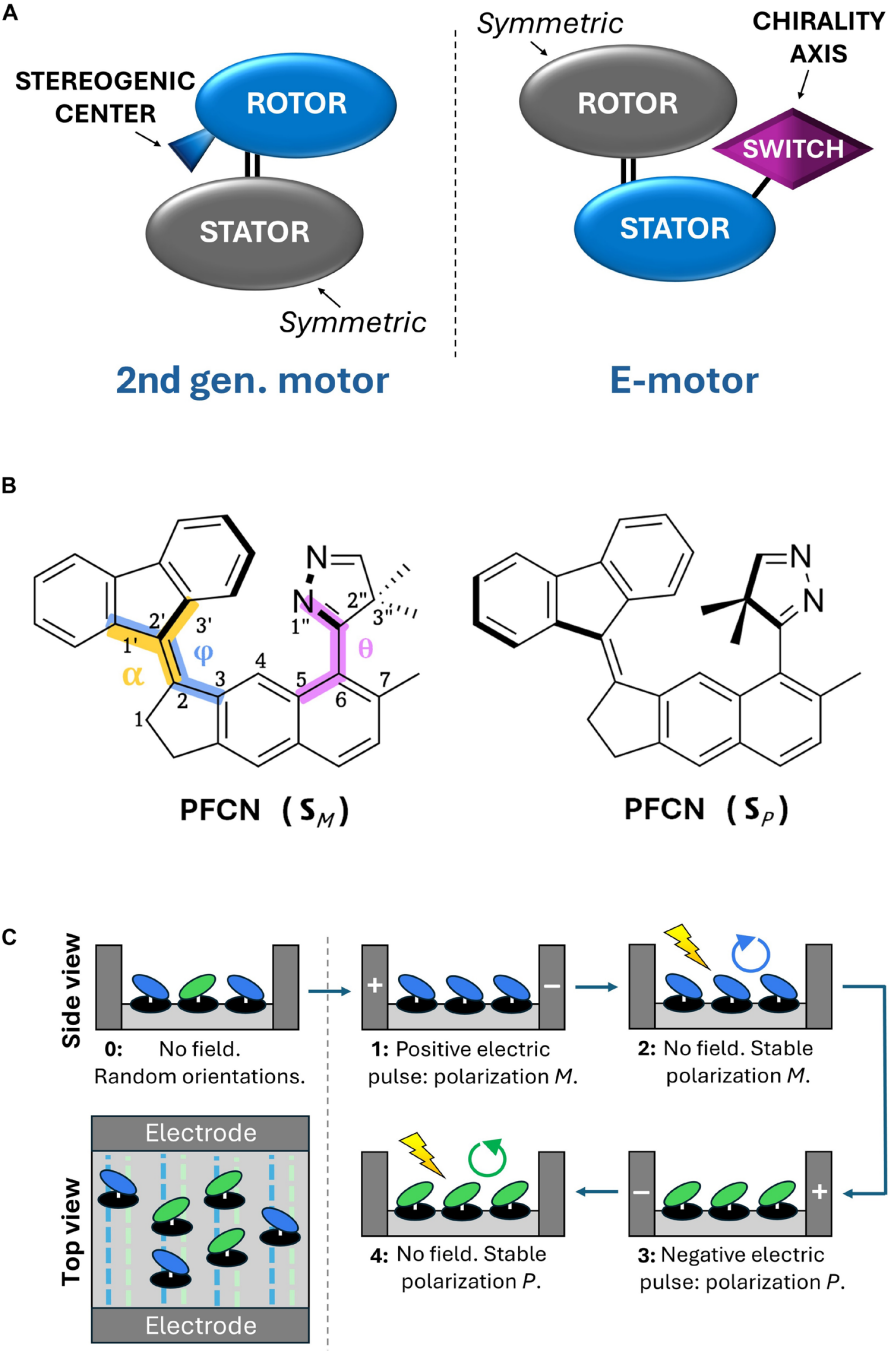
Electric field switchable motor structure. (**A**) Functional structure comparison between a typical second-generation motor and the switchable E-motor proposed in this work. (**B**) Chemical structure of the investigated PFCN system representing the E-motor class in its two stable forms of different chirality: **S***_M_* and **S***_P_*. The three marked dihedral angles allow us to track key structural changes of the operating motor: φ ≡ ∡ 1′-2′-2-3 (rotation of the rotor, in blue), θ ≡ ∡ 1″-2″-6-7 (rotation of the switch, in purple), α ≡ ∡ 2′-1′-3′-2 (rotor pyramidalization, in yellow). (**C**) Working principle and proposed arrangement of an array of electric field controlled molecular rotary motors.

In the present work, we propose a molecular rotary motor, the E-motor, whose direction of rotation can be controlled postsynthetically and on demand, by an external electric field switching pulse. The designed system and its working principle have been formulated on the grounds of quantum chemical and nonadiabatic molecular dynamics (NAMD) simulations. Our conceptual starting point is the second-generation motor structure, which we redesign by exchanging the point stereogenic center for a rotating, switchable unit, eventually achieving the motor’s axial chirality dependence on the orientation of the newly added switch (Fig. 1). The proposed switch is electric field sensitive: It characterizes with a large electric dipole moment, allowing for its orientation to be controlled with an external electric field. At the same time, thanks to the optimized steric repulsion between the switch and the principal framework of the motor, after the switch orientation is set with the electric field pulse, it is thermally (and optically) stable. Eventually, the motor can undergo a unidirectional, optically driven rotation in the absence of the field. It should be stressed that the discussed effect requires control of the motor orientation with respect to the external electric field source; thus, it is suited for controlling chirality of motors deposited on a surface (*29*–*32*) or possibly incorporated into a covalent or metal organic framework (*33*, *34*). Moreover, to assure all-*M* or all-*P* chirality of a manifold of surface-mounted motors, their stator components need to be aligned, e.g., in a way proposed in Fig. 1C, which could be achieved by proper functionalization of the surface by chemical or photolithographical means (*35*–*40*). Last, we would like to underline that, although in this work we provide numerous comments regarding the practical aspects of operation of an exemplary E-motor system, the primary aim of our study is to introduce the general concept of the E-motor architecture and its working mechanism, and the precise evaluation of the specific systems’ properties, including their interaction with the environment, will likely call for more extended theoretical and experimental studies in the future.

## RESULTS

### Detailed structure description of the PFCN motor

To illustrate the proposed motor design with a specific chemical realization, in this work, we investigate by means of quantum chemical methods a (9*E*)-9-(13-methyltricyclo[7.4.0.0^3.7^]trideca-1,8,10,12-tetraen-4-ylidene)-4b,8a-dihydro-9*H*-fluorene molecule (PFCN), whose chemical structure has been shown in Fig. 1. The proposed motor is composed of three distinguishable parts: (i) a symmetric rotor, (ii) a stator, and (iii) a polar switching unit.

The used rotor is made of a flat fluorene moiety, often employed as a stationary unit in the second-generation motors (*18*, *30*, *41*). It characterizes with a *C*_2*v*_ symmetry, and hence, its rotation by 180° leaves the system unchanged from a chemical point of view. Eventually, for a fixed-chirality operation cycle, the proposed motor is bistable, in similarity to its second-generation parent. Furthermore, the rotating fragment high symmetry assures that it does not contribute to the total system electric dipole moment.

The chosen stator, specifically its indane part, also resembles the rotating component popularly used in the first- and second-generation motors (*21*, *42*). The key difference is the lack of a methyl group at the cyclopentene ring, which, in traditional chiral motors, gives origin to the point stereogenic center. Another difference is addition of an extra toluene ring at the other side of the stator. This added fragment provides a scaffold for the switching unit. What should be noted is also an important role of the end methyl group within the toluene fragment, which protects the thermally activated switch from undesired rotation about the single C─C bond in the absence of electric field.

Last, the new motor component, the switching unit, is added at the far end of the stator. In the studied system, it is composed of a five-member, pyrazole-like ring with a dimethyl substituent placed opposed to nitrogen atoms. Eventually, one arrives with a push-pull moiety characterized with a pronounced dipole moment, with the negative charge localizing at the nitrogens, and the positive charge localizing at the methyl side of the switch. On this occasion, it might be worth noting that the push-pull substitutions are often successfully applied in molecular motor systems, especially in the context of their light-absorption properties and rotation mechanism control (*43*–*46*). Moreover, our proposed polarized switching unit might be, to a certain extent, compared to a zwitterionic molecular switch reported in an early work by Melloni *et al.* (*47*) The steric bulkiness of the designed switching unit is also strongly asymmetric. Thus, after the switch adopts its most energetically favorable perpendicular orientation with respect to the stator plane, its interaction with the rotor gives rise to a preference for one of the two axially chiral isomers (*P* or *M*), depending on the switch orientation. Also, the switch orientation by itself can be described in terms of axial chirality. This puts the here proposed E-motor into contrast with the motor architectures known so far, in which the operation direction is controlled either by a point stereogenic center (first- and second-generation motors) or by a pseudostereogenic structural arrangement (third-generation motors), with a noticeable exception of the achiral-host–chiral guest switchable molecular rotor mentioned earlier (*26*). A general outline for the possible synthetic pathway for the PFCN system can be found in a dedicated section in the Supplementary Materials.

### Ground-state characterization of PFCN stable and metastable forms in the absence of electric field

Below, and in the following section, to investigate the PFCN properties, we apply a multireference semiempirical method, ODM2/MRCI-SD (*48*), which allows for a robust, fully quantum chemical treatment of the designed system’s electronic structure, both in the ground and in the excited electronic states. All technical details regarding the performed calculations can be found in the Materials and Methods section and in the Supplementary Materials accompanying this publication.

**Fig. 2.**
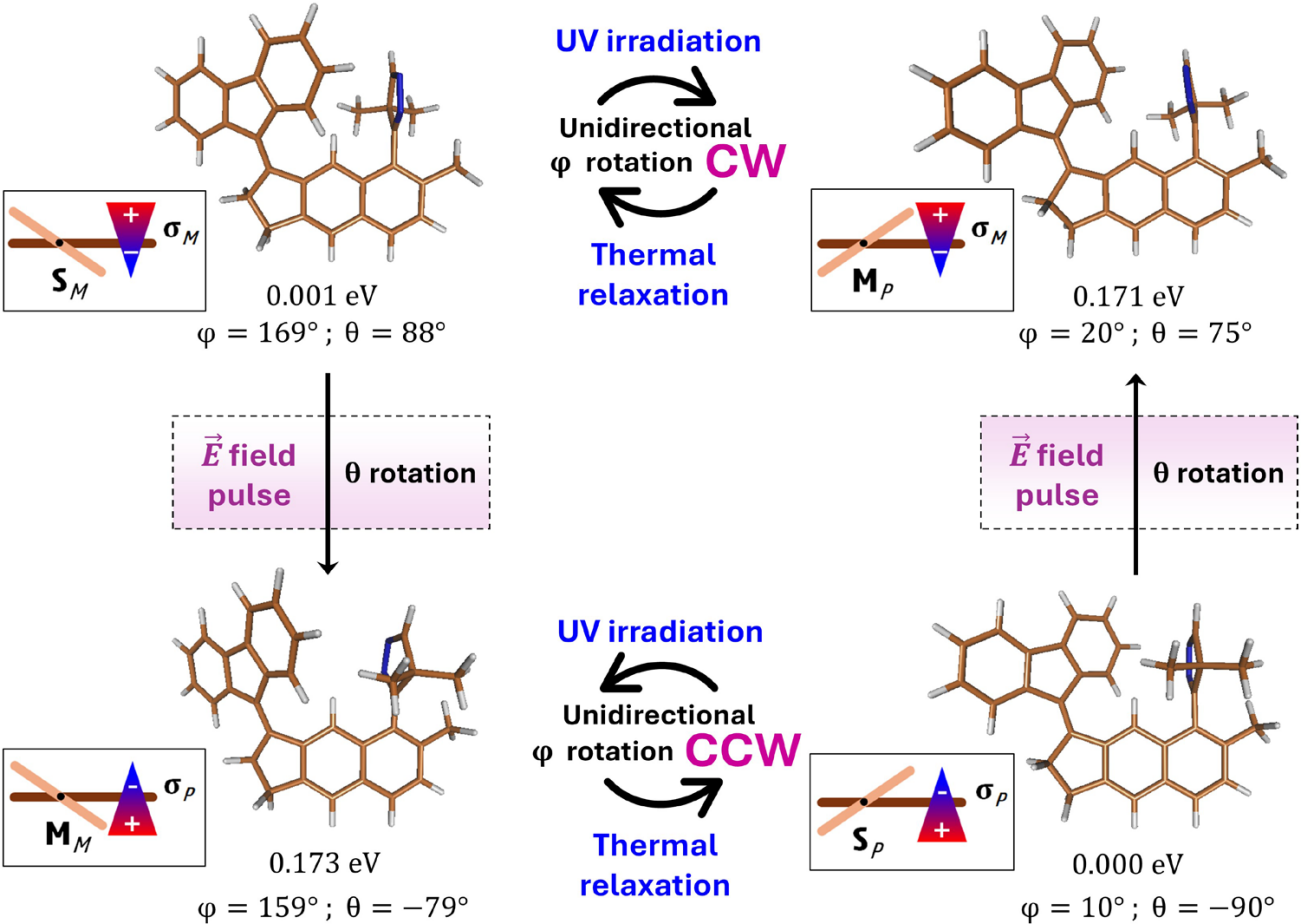
Operation cycle and switching scheme of PFCN. Displayed structures correspond to the ground-state stable PFCN forms, with indicated relative energies and characteristic dihedral angles, φ and θ, as defined in Fig. 1. The chirality of the motor forms (**S**/**M**) and the switching unit (σ) has been indicated in the inset pictures.

First, it should be pointed out that, in the ground electronic state, two mirror pairs of stable, **S**, and metastable, **M**, structures can be optimized: one pair for the CW and another for the CCW motor rotation, as illustrated in Fig. 2. It is also worth to note that the operation direction of the motor is governed by the axial chirality configuration of its **S** form, whereas the configuration of the **M** isomer participating in a given rotation cycle is always opposite. Upon a look at the optimized **S** and **M** structures and their characteristic features depicted in Fig. 2, one can immediately see the energy equivalence between the **S** and **M** forms of different chirality: an anticipated effect, given the chemical identity of these species. The ground-state relative energy of the metastable form is found at about 170 meV (3.9 kcal/mol), which resembles values reported for some known motors (*49*–*52*) and which assures thermodynamic equilibrium to be quantitatively shifted toward the stable isomer under the room temperature conditions.

Structure-wise, the key difference between the **S** and **M** isomers is the extent of the out-of-plane displacement of the rotor with respect to the stator, measured with dihedral angle φ, defined in Fig. 1. In the absence of steric hindrance, this angle should be close to 0°/180°, which would correspond to a flat arrangement, energetically preferred in the C═C bonded sp^2^ systems. At the same time, because of the steric repulsion between the rotor and the switching unit, this angle becomes distorted from planarity. Depending on which side of the switch the rotor moiety is interacting with, the repulsion strength varies, giving rise to different energies and different φ values of the **S** and **M** forms. This varying repulsion does not induce pronounced changes in the orientation of the switching unit, measured with dihedral angle θ, also defined in Fig. 1.

The key electric and ultraviolet-visible (UV-Vis) absorption properties of PFCN are shown in Table 1 on the example of the **S***_M_* and **M***_P_* forms. First, one should notice a pronounced ground-state electric dipole moment of both isomers, which is, to a great extent, preserved upon excitation to the two lowest-energy singlet excited states, *S*_1_ and *S*_2_. It should be also noted that, in both forms, the *S*_1_ state is the bright state, which can be reached with the UV light irradiation of about 3.75 eV (330 nm) and whose electronic configuration differs from a closed-shell ground-state configuration predominantly by the highest occupied molecular orbital (HOMO) → lowest unoccupied molecular orbital (LUMO) electron excitation. On the contrary, the *S*_2_ state, which requires excitation energy of roughly 4.20 eV (295 nm), shows a dominant HOMO-1 → LUMO excitation character and negligible oscillator strength. Upon inspection of the relevant orbital plots shown in the Supplementary Materials, it can be concluded that the *S*_1_ state follows the typical pattern known for unsubstituted overcrowded alkene motors, in which the lowest excited state is strongly delocalized, with clear ππ^*^ character and in which the initially double C═C bond between the rotor and the stator acquires a single-bond nature after the excitation. The *S*_2_ state, on the other hand, has a strong rotor-to-stator charge transfer (CT) character, which is likely responsible for the low *S*_0_ → *S*_2_ absorption strength.

**Table 1.** UV-Vis absorption properties of the S_M_(S) and M_M_(M) isomers, determined at the ODM2/MRCI-SD level of theory. *E*_ex_, *S*_0_ → *S*_*x*_ excitation energy; *f*, transition oscillator strength; El. config., dominant orbital contributions to the electronic transition; ∣μ∣, state electric dipole moment.

S	*E*_ex_ (eV)	*f*	El. config.	|μ| (D)
*S*_0_	–	–	–	4.65
*S*_1_	3.770	0.4641	H-L (91%)	4.20
*S*_2_	4.169	0.0052	H-1-L (85%)	5.81
**M**	***E***_**ex**_ **(eV)**	***f***	**El. config.**	∣μ∣ **(D)**
*S*_0_	–	–	–	4.38
*S*_1_	3.765	0.4788	H-L (91%)	4.28
*S*_2_	4.204	0.0050	H-1-L (84%)	5.31

Above-discussed PFCN key ground-state and UV-Vis absorption features have also been confirmed at the ab initio level of theory: Results of these calculations can be found in the Supplementary Materials. It should be noted that, although a very good agreement of the ground-state energies and structural parameters has been observed, the electronic excitation energies obtained at the ODM2/MRCI level of theory are systematically blue shifted with respect to the ab initio data. This effect is known for the used method and stays within its typical error of up to 0.5 eV (*53*). Eventually, in the following discussion, the evaluated total system energy after its optical excitation should be treated as an upper-bound limit.

### Potential energy landscape of a full PFCN rotation cycle

**Fig. 3.**
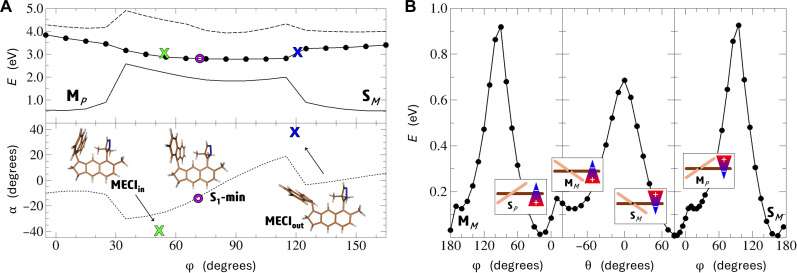
Potential energy profiles along the rotor (φ) and the switch (θ) rotation coordinates calculated in the absence of the electric field. (**A**) Rotor rotation during the optical step. Top: Energies calculated at PFCN structures optimized in the *S*_1_ state (solid line: *S*_0_; full circles: *S*_1_; dashed line: *S*_2_). The blue and green crosses show the positions of the optimized MECIs, and the purple circle marks the optimized *S*_1_ minimum. Bottom: Correlation between the rotor pyramidalization (α) and rotation dihedral angles (φ) along the optimized *S*_1_ profile and at the identified key structures. (**B**) Ground-state potential energy profiles calculated along the rotor rotation coordinate (left and right panels) and for the PFCN chirality switching (middle panel). The left and right panels correspond to the *CCW* and CW motor rotation cycles, respectively.

As has been already indicated, the most straightforward comparison of the proposed motor’s predicted operation mechanism can be made to that of the second-generation motors. Namely, for a given rotation chirality set by the orientation of the switch, the system starts with an optical *S*_0_ → *S*_1_ excitation of its stable isomer, **S**, initiating the so-called “optical” step of the rotation cycle. From that moment, the PFCN structure begins to evolve on the first excited-state potential energy surface (PES), which can be followed with the reaction path profile shown in Fig. 3A. Thanks to the maintained overcrowded alkene character, PFCN is expected to adiabatically relax on the *S*_1_ PES from the **S** Franck-Condon region toward the area of small *S*_1_/*S*_0_ energy gap values, where the central C=C bond between the rotor and the stator adopts close-to-perpendicular orientations. Just as in standard molecular motors, this rotation is driven by the topography of the *S*_1_ state PES, which, in turn, can be explained by system’s tendency to lower the energy of the antibonding LUMO orbital, populated in the *S*_1_ state. Two similar yet unequal minimum energy conical intersections (MECIs) have been found along this route, marked in Fig. 3A as **MECI**_**out**_ and **MECI**_**in**_. Both characterize with perpendicular rotor orientation and strong pyramidalization of the top carbon atom participating in the C=C bond but differ in the direction of this pyramidalization, with **MECI**_**in**_ showing slightly lower adiabatic energy of 3.041 eV versus 3.120 eV for **MECI**_**out**_. Both energies lie well below the initial *S*_0_ → *S*_1_ excitation energy, making the identified MECIs energy accessible. Eventually, it can be expected that, once the system arrives in their vicinity, efficient relaxation to the ground electronic state should take place, similarly as in the second-generation motors (*54*–*56*). After landing on the *S*_0_ PES, the motor can either come back to the starting **S** isomer or continue rotation toward the **M** form (right panel of Fig. 3B). Although different factors may affect the individual photoexcited motor fate, due to the presence of a large energy barrier separating the starting **S** and photoformed **M** isomers along the rotor rotation reaction coordinate, an incident value of this coordinate at the moment of the *S*_1_ → *S*_0_ hopping is likely to play an important role. In the case the rotation is continued, the vibrationally hot system encounters only a minimal energy barrier of about 110 meV (2.5 kcal/mol) to complete the final, so-called “thermal’ step” of the rotation cycle, reaching the product **S** form, chemically equivalent to the starting point. The expected, only short-time population of the metastable form shows some resemblance of the PFCN operation cycle to that of the two-stroke second-generation motor, recently proposed and investigated by theoretical and experimental means by Olivucci and co-workers (*57*)

Having said all this, we would like to draw the reader’s attention back to the problem of the system chirality. The reaction path shown in Fig. 3A and in the right panel of Fig. 3B illustrates the CW rotation of PFCN, sequentially populating the **S***_M_* → **M***_P_* → **S***_M_* structures. To reverse the motor rotation direction, one would need to inverse the axial chirality configuration of the **S** isomer. Having in mind that the **S** and **M** forms participating in the same rotation cycle characterize with the opposite configurations, this problem is equivalent to reversing the relative stability of these forms. This effect can be achieved through rotation of the PFCN switching unit, i.e., through inversion of its own axial chirality configuration, as illustrated in the central panel of Fig. 3B. Upon such rotation, the **S***_M_* isomer transforms into **M***_M_*, which is then expected to undergo quick thermal relaxation to form the **S***_P_* structure. Once formed, **S***_P_* can participate in the reversed direction, i.e., in the CCW motor operation cycle whose ground-state potential energy landscape, shown in the left panel of Fig. 3B, mirrors that of the CW rotation discussed previously.

In the absence of an external potential, the switching unit rotation requires substantial amount of energy: about 680 meV (15.7 kcal/mol). This value is about 25% smaller than the energy barrier protecting the system from the ground-state **S** → **M** forward rotation, corresponding to the tall peaks in Fig. 3B: 918 meV, equivalent to 21.2 kcal/mol. Eventually, the spontaneous reversal of the motor’s chirality appears unlikely at room temperature. At the same time, taking into account the possible temperature rise in the course of the motor photodriven operation or, on the contrary, in case the E-motor needed to operate in the much lower temperature regime, certain tuning of the PFCN chirality switching barrier might become necessary. In this respect, it should be pointed out that the modular structure of the proposed E-motor allows for almost independent control of the switch rotation barrier with chemical substitution at site 7 (Fig. 1), allowing its adjustment to one’s individual needs, as has been illustrated with few examples in the Supplementary Materials.

### Electric field control of the motor chirality

The large PFCN dipole moment originates primarily from the presence of the strongly polarized switching unit, whose electronic structure is shaped by push-pull effects induced by the dinitrogen and dimethyl substituents. Eventually, the total dipole moment aligns almost perfectly with the direction of the switch, which opens possibility for its manipulation with an external electric potential. It might be of worth to note here that, in the past, the static or rotating electric fields have been successfully used in numerous cases to power and to control rotating molecular systems (*58*–*61*).

In this respect, a simple picture of an electric dipole, $\vec{\mu }$, undergoing reorientation in the presence of an electric field, $\vec{E}$, might provide a good conceptual starting point. With the general expression for the dipole’s energy under field conditions, $E=-\vec{\mu }\cdot \vec{E}$, and given the perpendicular orientation of the switch in both PFCN stable forms, **S** and **M**, one can expect that the energy of one of the forms should increase whereas the other’s should decrease upon application of an electric field in a direction perpendicular to the stator plane. At the same time, the absolute potential energy at the maximum of the switch rotation barrier (middle panel of Fig. 3B) should not change much as this point corresponds to the null value of the $\vec{\mu }\cdot \vec{E}$ scalar product. Eventually, one can not only manipulate the PFCN isomer energies but also control the relative energy barrier for the switch rotation, i.e., the amount of energy necessary to reverse the motor operation direction.

**Fig. 4.**
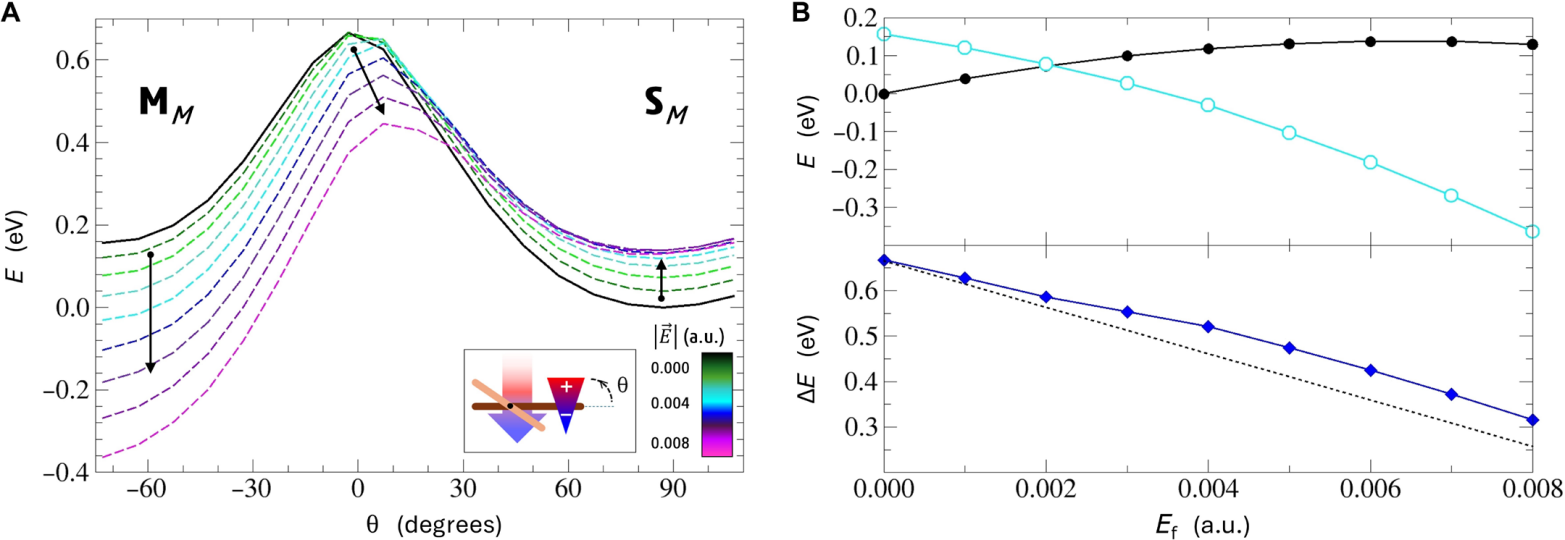
Electric field impact on PFCN key form energies in the ground electronic state. (**A**) Adiabatic potential energy profiles calculated between the **S***_M_* and **M***_M_* forms along the switch rotation coordinate at varying electric field intensities. (**B**) Extracted energies of the **S***_M_* (black full circles) and **M***_M_* (cyan empty circles) isomers (top panel) and **S***_M_* → **M***_M_* switching barriers (blue diamonds), along with the linear electric field stabilization function predicted with the dipole model (black dotted line) (bottom panel).

In Fig. 4 (left panel), we show potential energy profiles for the switching unit rotation in the presence of the external electric field of varying magnitude, calculated at the density functional theory (DFT) level (methodological details of these calculations are disclosed in the Supplementary Materials). In the studied case, the field is oriented perpendicularly to the stator, and its direction has been chosen so as it stabilizes the **M***_M_* isomer and destabilizes the **S***_M_* motor form. In the right panel of Fig. 4, key energy values associated with the switch rotation have been extracted for an easier reference.

First and foremost, upon inspection of Fig. 4, it can be noticed that expected changes of the key motor forms’ energies formulated on the grounds of the simple dipole model are generally confirmed, especially in the regime of small field intensities. With the increasing field strength, one can observe continuous lowering of the **M***_M_* energy, whereas the **S***_M_* energy undergoes a noticeable rise, which leads to intended reversal of the **S** and **M** forms stability. These changes are accompanied with a monotonic decrease in the **S** → **M** energy barrier. Eventually, when the barrier drops to the level comparable to thermal energy available to the system, a spontaneous **S***_M_* → **M***_M_* → **S***_P_* transformation is anticipated, realized through the switching unit rotation followed by a thermal rotor relaxation.

Upon a closer look at data shown in Fig. 4, one can notice that, although the observed changes’ trends follow the expectations, their quantitative evolution with increasing field strength diverges from the linear behavior predicted on the grounds of the dipole model. First of all, after initial destabilization under the weak electric field, the **S** form energy ceases to increase for field intensities greater than 0.005 atomic units (a.u.). Second, although, for weaker fields the energy maximum along the switch rotation path does not undergo noticeable changes, at $\left|\vec{E}\right|$ of about 0.004 a.u., lowering of the barrier starts to be observed. These findings indicate that, at an intensity of about 0.004 to 0.005 a.u., the electric field becomes strong enough to make the higher-order electric effects (such as molecular polarizability) dominate over the linear (dipole) effect in the investigated PFCN system. Nevertheless, the resulting combination of effects leads to promotion of the desired electric field–induced chirality inversion. Moreover, it should also be underlined that the investigated electric field intensity range of up to 0.008 a.u. corresponds to roughly 4 V/nm, which falls well within technical capabilities of the dedicated equipment used, e.g., in electrocatalytic studies (*62*).

### Real-time simulations of the PFCN motor performance

**Fig. 5.**
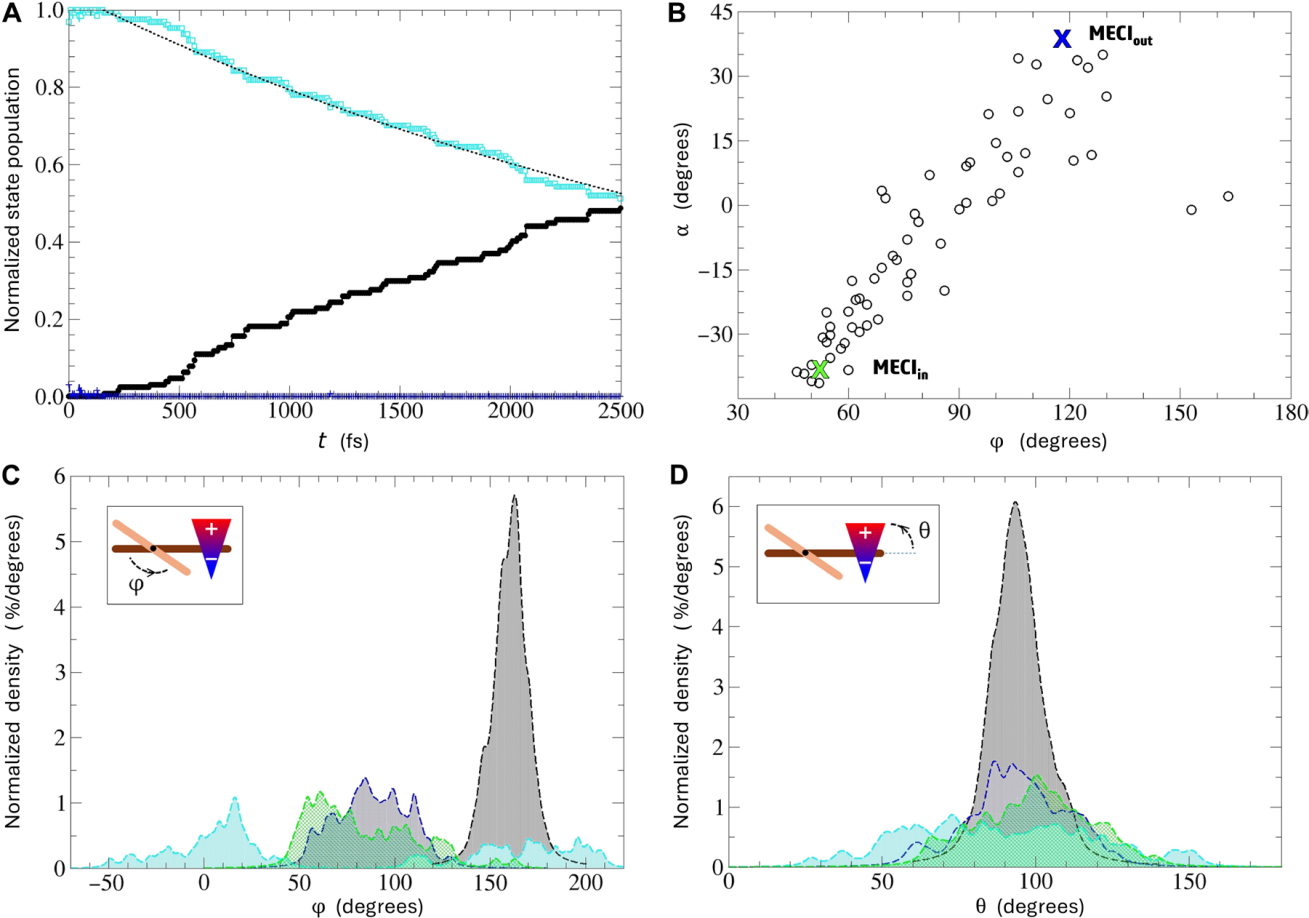
NAMD simulations of the PFCN motor operation. (**A**) Normalized mean electronic state population evolution in NAMD. Black full circles: *S*_0_; cyan empty squares: *S*_1_; blue crosses: *S*_2_; black dotted line: exponential fit to the mean *S*_1_ population decay. (**B**) φ/α dihedral angle correlation plot at *S*_1_ → *S*_0_ hopping points in NAMD simulations. The green and blue crosses mark positions of the optimized MECIs. Kernel density plots for φ (**C**) and θ (**D**) dihedral angles in the NAMD simulations. Black: initial distribution; green: distribution at *S*_1_ → *S*_0_ hopping points; cyan: final distribution from relaxed trajectories (finished in the *S*_0_ state); blue: final distribution from unrelaxed trajectories (finished in the *S*_1_ state).

Last, to ultimately confirm the molecular rotary motor character of the proposed PFCN system, we have performed NAMD simulations for the photoexcited **S***_M_* form relaxation, details of which have been described more extensively in the Supplementary Materials. In the course of the simulations, an ultrafast, single-exponential decay of the excited-state population has been observed (Fig. 5A), with fitted half-lifetime of about 3.6 ps, which stays in line with timescales observed in some second-generation motors (*55*, *63*). Analysis of *S*_1_ → *S*_0_ relaxation structures (Fig. 5B) revealed that the leading deactivation mechanism involves funneling through the lower-energy **MECI**_**in**_ conical intersection. In addition, some more extended relaxation area has been also observed between **MECI**_**in**_ and **MECI**_**out**_, corresponding to the central, low energy gap *S*_1_ PES region identified previously along the optical step motor reaction path (Fig. 3).

To have a notion of the temporal system evolution along the predicted reaction paths, in Fig. 5 (C and D), we have also shown kernel density plots for the two PFCN characteristic dihedral angles: φ and θ. Focusing on the rotor evolution first (Fig. 5C), one can observe that the initial, well-concentrated distribution centered around the **S***_M_*-optimized φ value consistently evolves toward the perpendicular rotor/stator orientation. After reaching this region, about half of the simulated NAMD trajectories successfully relax to the ground electronic state within the time span of the performed simulations. After the relaxation, trajectories split into two packets: one, continuing rotation toward the **M***_P_* form, and another, returning to the starting **S***_M_* structure, which stays in agreement with the known behavior of molecular motor systems. Quantitatively, in our simulations, 34 of 60 trajectories that relaxed to the *S*_0_ state continued the *M*-direction rotation. Eleven of them managed to go even further, beyond the metastable isomer, overcoming the thermal barrier to form the rotated **S***_M_* isomer.

Concluding analysis, essential for assessing PFCN eligibility to act as a switchable unidirectional rotary motor, is checking the switching unit stability in the course the optical motor operation. This can be done by looking at the switch direction changes in the course of the photodynamics simulations. Upon inspection of Fig. 5D, one can immediately spot a well-picked distribution of initial θ values, confirming the *M* chirality of the starting stable isomer. As the dynamics proceeds toward the *S*_1_ → *S*_0_ relaxation region on the *S*_1_ PES, gentle broadening of this peak is observed. After the relaxation, this effect becomes even more pronounced, which can be ascribed to increasing kinetic energy accumulated in the system. At the same time, the span of dihedral angles visited by the switching unit in the course of the dynamics never comes near a point at which its orientation could be flipped, which would result in the instability of system’s chirality. Eventually, we conclude on promising PFCN properties for application as an electric field switchable molecular rotary motor.

## DISCUSSION

In summary, we have proposed a molecular rotary motor architecture, in which the motor operation direction can be switched in situ, without the need for chemical modification of the system structure. This is achieved by application of an external electric field pulse and is intended to control chirality of motors deposited on a surface. Our study relies on quantum chemical calculations and NAMD simulations performed for a specific chemical system, PFCN, designed to provide illustration for the proposed motor type. On the grounds of the obtained results, we show that the model system’s chirality and hence its operation direction depends on axial chirality of a covalently bound polar switching unit, which can be efficiently controlled with an external electric field. At the same time, the proposed system manifests all characteristic photophysical properties of a unidirectional molecular motor, and its chirality is preserved, i.e., it is thermally and optically stable during the regular motor operation in the absence of the field.

Although the presented results comprise data obtained for an isolated molecular system, the practical realization of the proposed switchable motor will additionally involve (i) its deposition on a surface, (ii) precise control of the molecular alignment with respect to the external electric field, and (iii) inclusion of possible interactions with the environment. The first problem will likely call for further E-motor functionalization with suitable molecular linkers, as has been done before for other motor systems (*29*–*32*). In the E-motor case, however, to allow for the control of its spatial orientation with respect to the field, which concerns the second problem, the usage of two different linkers is necessary, along with a proper prestructurization of the surface, in which each of the linkers could bind with a dedicated site (*35*–*40*). Last, if the motor was to operate in solution, low-polarity solvents should be considered to avoid the strong interaction between the solvent and the switching unit on the one hand and to minimize the electric screening of the externally applied electric field on the other. Although detailed investigation of all these effects falls beyond the intended scope of the present study, some further analysis and guidance on that matter, along with preliminary results, can be found in the Supplementary Materials.

## MATERIALS AND METHODS

All results reported in the manuscript have been obtained with theoretical chemistry methods. The electronic structure calculations presented in the main text were performed with two approaches: with the semiempirical ODM2/MRCI method (structural optimizations, relaxed potential energy profiles scans, absorption properties determination, and NAMD simulations) and with the DFT (potential energy profiles calculations in the presence of external electric field).

### Semiempirical calculations

The ODM2/MRCI method (*48*), developed by Thiel *et al.* and implemented in the mndo2020 software (*64*), is a semiempirical approach combining orthogonalization and dispersion corrections with a multireference configuration interaction framework, restricted in our study to single and double excitations (the MRCI-SD variant). We use the orbital active space (AS) containing 10 electrons distributed in 11 orbitals (10,11), with the molecular orbitals optimized for the lowest-energy open-shell configuration, i.e., configuration in which the HOMO and LUMO orbitals are singly occupied (H-L). The plots of the AS orbitals used in the semiempirical calculations can be found in the Supplementary Materials. In the multireference configuration interaction procedure, three reference electronic configuration determinants were used: the one used for the orbital optimization (H-L) and the two closed-shell configurations that can be built therefrom (i.e., H-H and L-L).

The ODM2/MRCI method has been chosen for its great calculation cost efficiency, allowing orders of magnitude savings when compared to more standard ab initio approaches. At the same time, the accuracy of ODM2/MRCI (and of the closely related OM2/MRCI approach) has been proven sufficient to reproduce and rationalize experimental findings and/or higher-level theoretical results in numerous prior photochemical and photophysical studies on medium-size organic systems (*65*–*68*), including examples of molecular motors (*56*, *69*–*73*). Eventually, thanks to the semiempirical ODM2/MRCI approach robustness combined with the reasonable accuracy, it was possible to perform for the studied PFCN system not only the structural-optimization and excited-state reaction paths calculations but also the costly real-time NAMD simulations, which deliver unique insights into mechanisms, rates, and efficiencies of photoinduced processes at the nanoscale.

### DFT calculations in the presence of electric field

The part of the study, in which we explicitly investigated the electric field impact on the ground-state potential energy profiles, was conducted using the DFT, with the B3LYP hybrid exchange-correlation functional (*74*–*76*) and the def2-SVP basis set (*77*). The Grimme dispersion correction with Becke-Johnson damping, D3-BJ, was applied to assure reliable description of the noncovalent interactions (*78*, *79*). It should be pointed out that, although the B3LYP functional is generally not suited for describing the electronic excited states in molecular motors, especially those of partial CT character, it usually performs very well for the ground-state structures and the electronic properties determination (*72*, *73*, *80*, *81*). All B3LYP/def2-SVP calculations have been performed with the turbomole suite of programs (rev. V7-7-1) (*82*).

To investigate the PFCN behavior in the presence of electric field, it was first necessary to determine the electric field direction perpendicular to the stator plane. To this end, we applied the following protocol, also illustrated in the Supplementary Materials: (i) definition of two vectors, ${\vec{v}}_{1},{\vec{v}}_{2}$, connecting atoms selected from the stator’s central (naphthalic) fragment, which span the stator plane; (ii) algebraic determination of the vector normal to the stator plane; and (iii) rescaling of the normal vector, according to the magnitude of the applied electric field, $\vec{E}$.

### Computational settings for the dynamics simulations

For the propagation of the NAMD, Tully’s fewest switches surface hopping (*83*, *84*) algorithm was used, as implemented in the mndo2020 package. To this end, first, a 3-ps thermalization Born-Oppenheimer molecular dynamics (MD) was run under the canonical ensemble with the temperature set at 298 K. In this part, a Nosé-Hoover thermostat (*85*) with a chain length of 10 and characteristic time of 0.05 ps was used. Subsequently, a microcanonical ensemble production run was propagated for another 2 ps. Of the structures generated during this second MD step, 300 points were selected randomly for the initial condition filtering procedure. The filtering was performed following a standard approach (*86*), in which a transition energy window of 3.70 ± 0.25 eV and the optical transition probability criteria were applied.

Eventually, a set of 127 NAMD trajectories was run for a maximum time of 2.5 ps. In these simulations, gradients and nonadiabatic couplings (NACs) were computed analytically on the fly at the ODM2/MRCI level. Electronic motion was propagated using the unitary propagator evaluated at the middle point (*87*). The adaptive timestep algorithm (*88*) was also used, with an absolute energy change threshold set to 0.01% and with the maximum number of step reductions of 10. Decoherence was accounted for using the Shenvi-Subotnik’s parameterless correction (*89*). After a successful interstate hopping, nuclear velocities were rescaled along the direction of the NAC vector. All frustrated hops were rejected, with reversion of the velocity component parallel the NAC vector. In all performed MD and NAMD simulations, a 0.5-fs timestep for the nuclear and 0.0025-fs timestep for the electronic structure propagation were used, with continuous orbital-phase tracking between the consecutive dynamics steps.
