# Supplementary material for: Unidirectional molecular rotary motor with remotely switchable rotation direction
Source: Sci Adv. 2025 May 16;11(20):eadt8008. doi: 10.1126/sciadv.adt8008 (PMC13109948; doi:10.1126/sciadv.adt8008)
Supplement: Supplementary file 1 — Supplementary Text Figs. S1 to S12 Tables S1 to S13 [file sciadv.adt8008_sm.pdf]

Science Advances
AAAS

Supplementary Materials for

Unidirectional molecular rotary motor with remotely switchable rotation direction

Kamil Szychta *et al.*

Corresponding author: Joanna Jankowska, jjankowska@chem.uw.edu.pl

Sci. Adv. **11**, eadt8008 (2025)
DOI: 10.1126/sciadv.adt8008

This PDF file includes:

Supplementary Text
Figs. S1 to S12
Tables S1 to S13

Supplementary Text

Supplementary ab initio calculations results

To benchmark the ODM2/MRCI results, several series of calculations at various ab initio levels of theory were performed. Firstly, we reoptimized the key ground-state forms of PFCN at the DFT B3LYP-BJ level. Secondly, UV-Vis absorption properties of these forms were checked with: quasi-degenerate second order n-electron valence state perturbation theory (QD-NEVPT2), (*90*) time-dependent density functional theory (TD-DFT) with the long-range and dispersion corrected hybrid exchange-correlation functional wB97x-D, (*91*) Tamm-Dancoff time-dependent density functional theory (TDA TD-DFT) with the wB97x-D and with the BJ-corrected Coulomb-attenuating B3LYP functional (CAM-B3LYP-BJ), (*92*) and with the mixed-reference spin-flip time-dependent density functional theory (MRSF TD-DFT; **S**-form only) combined with CAMB3LYP-BJ. In these calculations, we have employed the Karlsruhe double-ζ (def2-SVP) and triple-ζ (def2-TZVP) (*93*) basis sets with polarization functions (in the QD-NEVPT2, DFT, TD-DFT, and TDA TD-DFT calculations), and the polarized Dunning double-ζ cc-pVDZ (*94*) basis set (in the MRSF TD-DFT calculations).

In the next step, relaxed potential energy profile (PEP) scans along the operation coordinates of the studied **E-motor** were performed, both in the ground, and in the excited electronic state (respectively, at the DFT and MRSF TD-DFT level of theory). Furthermore, results of an unrestricted S_1 state optimization at the MRSF TD-DFT and ODM2/MRCI level were compared. Finally, certain effects of solvation have been evaluated with use of the Conductor-like Screening Model (COSMO).

Results of all these calculations are shown and discussed below. All DFT-level structural optimizations were performed with the TURBOMOLE suite of programs, (*82*) the TD-DFT and TDA TD-DFT absorption properties in vacuum were evaluated with the GAUSSIAN 16 software (rev. C.01), (*95*) while TD-DFT absorption calculations in solvent were performed with TURBOMOLE. The MRSF calculations were conducted using the GAMESS code. (*96*) For the QD-NEVPT2 calculations, the ORCA software (version 5.0.4) was used. (*97*)

Ground-state benchmark calculations

To benchmark the ODM2/MRCI performance for PFCN ground-state structures, firstly we reoptimized the **S** and **M** forms at the B3LYP-BJ/def2-SVP level of theory, which was also used for the PFCN PEP scans under electric-field conditions. Overall, very good agreement of results obtained with both approaches is observed (table S3): both in terms of relative energies, and for the characteristic structural features; for definition of the analyzed dihedral coordinates, please refer to figure S2.

In the next step, to get an insight into the quality of the ground-state potential-energy description beyond the minimum-energy forms, PEP scans along the rotor (φ), and along the switch (θ) rotation coordinates were performed at the same level of theory, B3LYP-BJ/def2-SVP, as depicted in figure S3. The obtained energy barriers, again, match well the analogous values from the scan performed at the ODM2/MRCI level (Figure 3b in the main text), with the main difference being predicted barrierless **M**→**S** relaxation in the course of the thermal step which, however, does not alter the predicted motor operation mechanism in any noticeable way. For an easier reader's reference, all energy-barrier values have been extracted and are shown below, in table S4.

UV-Vis absorption properties

In Table 1 in the main text, one can find PFCN absorption properties determined using the ODM2/MRCI method, which have been here repeated for the reader's convenience (table S5). Additionally, in table S1 and in table S2 in the first Section of this Supplementary, one can find active space molecular orbitals plots for the **S** and the **M** form. As can be noticed, in both forms the bright $S_0 \rightarrow S_1$ transition possesses a local-excitation (LE) character (HOMO→LUMO), in contrast to $S_0 \rightarrow S_2$, which can be described as a dark charge-transfer (CT) state (HOMO-1→LUMO).

The PFCN absorption properties determined at the ODM2/MRCI level can be compared with corresponding ab initio results presented below. Firstly, in table S6 one can find vertical excitation energies and oscillator strengths evaluated at various levels of theory for the PFCN system, and for a popular second-generation motor for which theoretically-predicted absorption energy can be compared to the experiment (compound **4a**-H in Ref. (*41*)). As has been already indicated in the main text, upon inspection of the presented data one can observe a systematic blue-shift of the

ODM2/MRCI excitation energies for both motors with respect to the ab initio data and to the experimental value reported for **4a**H. This shift, although within the typical range expected for the semi-empirical OMx methods, (*53*) indicates that the total energy of the photoexcited PFCN system in the current study should be considered an upper-bound limit.

Secondly, in table S7 and in table S8 more detailed characterization at the TDA TF-DFT CAM-B3LYP-BJ/def2-SVP level of the two lowest-energy transitions in PFCN can be found. It can be observed, that for the leading $S_0 \rightarrow S_1$ transition, the obtained data are in very good agreement with the ODM2/MRCI results: the transition to the S_1 state occurs with high oscillator strength, and characterizes with energy of 3.59 eV, only slightly lower than the 3.77 eV predicted at the ODM2/MRCI level. Another similarity is the preserved character of both transitions: S_1 - LE, S_2 - CT. At the same time, one can observe slightly larger difference in predicted $S_0 \rightarrow S_2$ excitation energies: around 4.0 eV at the ODM2/MRCI level, and ~ 3.7 eV with TDA TD-TDFT. Such difference, however, stays within the mean excitation-energy error typical of the OM2-based methods (*48*) and does not alter the predicted motor operation mechanism in any way.

Finally, the absorption properties of the **S** form of PFCN calculated with the MRSF TD-DFT method, with CAM-B3LYP exchange-correlation functional and with the cc-pVDZ basis set, (*94*) are shown in table S9 and in table S10. At this level of theory, the energy gap between the bright S_1 state and the dark S_2 state becomes further reduced to only 0.08 eV. At the same time, the character of both transitions stays in full agreement with the prior results, obtained with ODM2/MRCI and with the TDA TF-DFT CAM-B3LYP-BJ/def2-SVP methods.

Characteristic features of the key forms relaxed in the S_1 state

In table S11 one can find relative energies and values of characteristic structural parameters of the key PFCN forms relaxed in the S_1 state: the S_1-state minimum, and the optimized S_1/S_0 minimum-energy conical intersection points (MECIs). For the S_1-state minimum, one can compare results obtained at the ODM2/MRCI and at the MRSF CAM-B3LYP/cc-pVDZ levels of theory. What can be observed is, firstly, a small down-shift of the S_1-minimum adiabatic energy at the MRSF level of theory and, secondly, observably smaller pyramidalization of the carbon atom linking the rotor and the stator fragments (the α dihedral value). At the same time, the S_1/S_0 energy gap predicted at the S_1-minimum structure remains similar.

Adiabatic potential energy profile scans

To examine perspectives for motor operation of PFCN upon the UV-irradiation (i.e., in the course of its optical operation step), the relaxed scans in the S_1 state were performed using the ODM2/MRCI and MRSF CAM-B3LYP/cc-pVDZ methods, results of which are shown in figure S4. In both cases, after the **S**-form excitation ($\varphi \approx 160^o$) to S_1, a spontaneous rotor rotation about the central double bond is expected, leading directly from the Franck-Condon region of the **S** form to the S_1-state local minimum, characterizing with strongly reduced S_1/S_0 energy gap. In the course of this rotation, the bright-S_1/dark-S_2 state swap can be also noticed at φ dihedral angle values of about 30° and 120°. This predicted structural and electronic evolution, as well as the overall PEP shape, resemble well typical characteristics of certain known second-generation molecular rotary motors. (*21, 98–100*)

Chemical-substitution control of the switch rotation barrier

The modular structure of the proposed **E-motor** opens possibilities for independent control of the switch rotation barrier with chemical substitution at site 7. Eventually, it becomes possible to tailor the switching-properties according to individual needs, without changing other key operational properties of the motor.

In figure S5, one can find ground-state PEPs along the rotor rotation (φ) and along the switch rotation (θ) coordinates calculated in the absence of the electric field for the PFCN system and its four 7-substituted derivatives. The calculations for the substituted motors have been performed at the ODM2/MRCI level of theory with the 10-in-11 orbital active spaces, in analogy to the original calculations performed for PFCN. It can be observed that, by interplay of the steric repulsion, substituent rigidity, and electrostatic attraction, it is possible to modulate the switch rotation barrier by virtually arbitrary energy shift, both in the upward and in the downward direction. At the same time, the ground-state $\mathbf{S}_M \rightarrow \mathbf{M}_P$ energy barrier, essential for the motor operation stability, remains unchanged.

Justification for the initial-condition filtering parameters

In figure S6, one can find a dynamically-averaged absorption spectrum (panel (a)) and an energy vs. oscillator strength correlation plot (panel (b)) for the PFCN system. The spectrum was built as a

sum of vertical transitions calculated at all 6000 points from the production, i.e., the microcanonical MD run. Each transition peak was broadened with a Lorentzian function with arbitrary full-width at half-maximum (FWHM) of 0.02 eV. Upon inspection of figure S6 (a), it can be observed that the PFCN absorption below 4.0 eV ($\lambda > 310$ nm) is dominated almost exclusively by the bright $S_0 \rightarrow S_1$ transition. With the data shown in figure S6 (b) it can be confirmed that the $S_0 \rightarrow S_1$ transition is well isolated from others in terms of energy and vibrational state-mixing. Eventually, with the employed choice of the excitation-energy window in the initial-conditions filtering procedure (3.70 ± 0.25 eV), one obtains unbiased, uniform set of the NAMD starting points, weightened by the $S_0 \rightarrow S_1$ absorption coefficient function of PFCN.

At the same time, it might be of worth to note that, due to its quantum-classical nature, the Born-Oppenheimer MD applied for the initial-conditions generation might underestimate the energy accumulated in the vibrational degrees of freedom of the studied molecule by neglecting the zero-point energy effects. (*101*) To, at least roughly, assess the possible impact of this effect on the PFCN motor photodynamics, following the strategy employed in the cited reference, (*101*) we have performed a high-temperature MD, with the initial thermalization run in 900 K. In figure S6 (c), we show for comparison a dynamically-averaged PFCN absorption spectrum obtained from the high-temperature run. As can be observed, compared to the reference room-temperature results shown in panel (a), the 900 K spectrum adopts a little broader and red-shifted shape, indicating possible population of structures which were not present in the lower-energy dynamics. At the same time, the actual energy downshift of the absorption peak center in the high-temperature spectrum is rather small (< 0.1 eV), resulting in a large overlap of the hypothetical 900 K absorption window with the one applied in our simulations. Eventually, we believe that the originally employed initial-conditions generation procedure should provide a reasonable approximation to modeling the photo-induced dynamics of PFCN.

Exponential-decay fitting procedure

In order to estimate the S_1-state relaxation time in the NAMD simulations performed for the PFCN **S** form, the S_1 state population decay has been fitted with a single-exponent function, as shown in figure S7. In this procedure, the t_0 parameter controlling the initial decay delay has been manually fixed at the value shown in the inset to improve the total stability of the fit.

Preparation of the kernel density plot snapshots

To have a notion of the PFCN structural evolution in time along the predicted relaxation path, in the main text in Figure 5 (panel c and d) we have shown kernel density plots for the two PFCN characteristic dihedral angles, φ, and θ, changing in the course of the NAMD trajectories. These graphs present snapshot, histogram-like distributions of the chosen internal-coordinate values for the indicated relaxation process stages. The smooth density functions were obtained by applying the Gaussian data broadening, with the FWHM set at 5°. The individual employed Gaussian functions were normalized in such a way, that integration of the initial kernel density distribution, built of the data from all 127 run trajectories, gives unity (100%).

Outline for the possible synthetic pathway for the PFCN system

The proposed possible synthesis of the target PFCN molecule, illustrated in figure S8, begins with the oxidation of 2,6-dimethylnaphthalene (**1**) using potassium permanganate to form a carboxylic acid. (*102*) This is followed by regioselective bromination at the 5-position of the naphthalene core to yield a brominated intermediate. (*103*) Subsequent reduction of the carboxylic acid to the alcohol using $LiAlH_4$ and bromination of the resulting hydroxyl group provides intermediate (**5**), which is a precursor for introducing the dimethylmalonate moiety. In the presence of a strong base, dimethylmalonate undergoes alkylation, forming dimethyl ester (**6**).

The ester (**6**) is then subjected to saponification and decarboxylation, yielding the carboxylic acid (**7**). To enhance the molecule's reactivity, the acid is converted to its corresponding acid chloride (8) using $SOCl_2$. The acid chloride then undergoes a Friedel-Crafts acylation in the presence of $AlCl_3$ and nitromethane, resulting in a key cyclized intermediate (**9**). It should be noted that, while the desired regioselectivity of Friedel-Crafts acylation may be achieved using polar solvent (nitromethane or nitrobenzene) and slow addition of $AlCl_3$ to the acylation mixture, i.e., by applying conditions similar to Perrier addition sequence, (*104*) a significant amount of undesired regioisomers is expected to form. (*105*)

The next step involves transforming intermediate (**9**) into a hydrazone derivative, enabling the subsequent Barton–Kellogg olefination. The Barton–Kellogg reaction is carried out using thiofluorenone and a diazo compound, the latter can be generated from the hydrazone derivative

with a hypervalent iodine reagent. (*106*) This reaction leads to the formation of a thiadiazoline, which is thermally unstable and rapidly eliminates nitrogen. The episulfide intermediate formed during this process is subjected to sulfur extrusion using triphenylphosphine (PPh_3), producing a coupled aromatic system (**10**).

A switching unit is introduced at this stage by lithiation and subsequent reaction with 2-(2-methyl-2-cyanoethyl)-1,3-dioxolane, yielding a protected intermediate. At the same time, it should be noted that the lithium-halogen exchange reaction may be associated with the formation of substantial amounts of byproducts: alternatively, Turbo-Gringard reagents and appropriate electrophile may be used. The final step involves deprotection and cyclization, where the diketone (**11**) reacts with hydrazine hydrate, affording the target molecule, PFCN.

Discussion on the surface-deposition effects

The deposition of motor molecules on a surface is most often achieved through addition of an organic scaffold (molecular linker) substituted to the stator part of the system, e.g., as illustrated in the review article by García-López. (*28*) The scaffold separates the motor from the surface, which helps to avoid the quenching, and to prevent the uncontrolled free rotation of the whole motor molecule: in particular the latter effect, essential for the present study, is achieved by application of two molecular linkers. With the proper substituent choice, one can control the level of strain applied to the stator part of the motor to minimize the surface-binding impact on its performance. (*29*)

The effect that could be potentially persistent, though, is the electron-donating / electron-withdrawing effect of the scaffold substituent. To assess its possible impact on the key properties of PFCN, we have calculated relaxed potential energy profiles scans in the ground and in the first excited electronic state for two model PFCN derivative systems which included organic linkers inspired by units used in experiments. (*29, 107*) These calculations have been performed at the ODM2/MRCI-SD level, with the same orbital active space as before, comprising 10 electrons distributed in 11 orbitals. The visualization of the designed systems and the results of these calculations are presented in figure S9. In panel (a) of figure S9 one can see chemical structures of the two PFCN derivatives: PFCN-sca-1 with two identical linkers capped with a thiol group, and PFCN-sca-2, in which one of the linkers has been replaced with an aliphatic chain containing an amide group; for the rationale for choosing these two substituents, please check the next paragraph.

Upon inspection of the profiles shown in figure S9 (b) it can be observed that the chosen scaffolds do not have a meaningful impact on the PFCN system properties, such as energy barriers and, more generally, the topography of the ground-state and lowest excited-state potential energy surfaces in the regions relevant for the motor rotation (optical and thermal step), nor for its switching.

Another, related problem is maintaining the precise alignment of the motor molecules, which is crucial for the electric-field control of their chirality. Such an alignment could be achieved by combining a proper choice of the linking substituents, and corresponding preparation of the surface, at which the motors are to be deposited. To this end, firstly, one should use two linkers, to eliminate the possibility of the rotation of the entire system during its optical operation, or the switching. Secondly, the linkers should characterize with different affinity to the surface, e.g., one should bind easily to a bare metal surface (like a thiol group), (*29*) while the other should exclusively bind to pre-functionalized sites (like a carboxylic group forming an amide bond with surface-mounted amine chains). (*107*) In this way, with a properly structured surface, a unique spatial orientation of motors could be achieved, as schematically shown in figure S10. While at the moment we are not aware of a practical realization of this exact arrangement, to the best of our understanding, the currently available surface functionalization methods do allow for preparation of setups similar to the one proposed herein. (*40, 108*)

Finally, it might be of worth to analyze the possible impact of an imperfect alignment of surface-deposited motor molecules on the efficiency of their field-induced switching. Firstly, let us consider a deviation from the parallel alignment of a motor with respect to the electric field which does not exceed 90 degrees. In such case, as illustrated in figure S11, the effect of the field on the relative energies of the isomers is weakened (smaller stabilization/destabilization of the **M/S** form, respectively), and the **S** $\rightarrow$ **M** transformation energy barrier undergoes an upward or downward shift, depending on the direction of the field aberrance. These observations stay in full accordance with the proposed simple model of the electric dipole interacting with an external electric field. On the other hand, if the **E-motor** molecule is rotated by more than 90 degrees, the effect of the applied external electric field for such a system will be reversed in the sense that a mirror motor chirality will be stabilized. Eventually, it can be predicted that the moderate deviation from the parallel alignment of motor molecules does not fundamentally preclude collective control of their chirality but should rather result in the need for application of stronger electric fields. However,

if the molecules' orientations are completely random, while the electric field can still be used to switch the chirality of the individual motors, the CW/CCW rotation direction will not be consistent between different systems.

Discussion on the effects of interaction with the environment

Another important effect that could impact the **E-motor** performance is its possible interaction with solvent. While, technically, the surface-deposited PFCN could operate in a solvent-free environment, one may try to make some predictions regarding its performance on a solvent-moistened surface. Given the fact that PFCN is an inherently polar system and is to be controlled with an external electric field, solvents characterized with low dielectric constants should be considered. In this regard, we had a look at the light-absorption properties of the **S** and **M** PFCN forms, and at the switching-unit rotation potential energy profiles in two solvents: hexane and DCM, included using the Conductor-like Screening Model (COSMO). At this stage, we would also like to note on the grounds of the experimental results available in the literature that, while in general the solvent polarity might have a pronounced impact on molecular motors' performance, (*46, 109*) in low-polarity (and low-viscosity) solvents, traditional motors' operation seems to be almost unaffected by the changing environment. (*45, 63*)

Firstly, in tables S12 and S13, one can find data illustrating the expected PFCN absorption in the presence of the two selected solvents, determined respectively at the ODM2/MRCI-SD (with AS including 10 electrons distributed in 11 orbitals) and at the TD-DFT wB97x-D/def2-TZVP levels of theory. Upon inspection of the data, it can be observed that the $S_0 \to S_1$ and $S_0 \to S_2$ transition energies remain practically constant when the environment changes from vacuum to hexane and DCM, which is accompanied with a slight increase of the respective transitions' oscillator strengths when moving towards the more polar environment. Importantly, the character of all transitions is fully retained.

Furthermore, as one can tell on the grounds of the switching-unit rotation profiles shown in figure S12, calculated in the absence of the electric field at the previously employed DFT B3LYP-BJ/def2-SVP level of theory, the energies of the relevant forms and the barrier height do not seem to change much in the presence of the chosen solvents. While modeling the effect of the solvent in the presence of the external electric field would call for applying an explicit-solvent approach (such

as QM/MM simulations) which is beyond the scope of the current study, our expectation is that the main effect in such case would likely be a reduction of the perceived electric field intensity due to the solvent dielectric screening (non-polar solvents) and/or due to field-induced reorientation of the polar solvent molecules. In both cases, this would result in an increase in the voltage required to switch the **E-motor's** chirality: in the first order, this increase would be proportional to the applied-solvent dielectric constant. As a final remark to this point, we would like to mention that, while we have not performed the excited-state potential energy profiles scans with the inclusion of the solvent, experimental data available for comparable existing second-generation motor in the gas-phase and in the nonpolar solvent suggest the effect low-polarity solvents is not expected to be strong. (*110*)

Cartesian coordinates of key PFCN structures

S_0**-opt:** PFCN (S) - ODM2/MRCI (10,11)

C	-2.908142	2.346441	-4.716728
C	-3.805590	1.973602	-3.721539
C	-5.195148	1.914436	-4.022875
C	-5.666501	2.088766	-5.313935
C	-4.733354	2.345990	-6.323102
C	-3.382216	2.505493	-6.022108
C	-3.617838	1.738784	-2.274532
C	-4.980151	1.722116	-1.698293
C	-5.925492	1.743037	-2.764879
C	-7.288265	1.650218	-2.533272
C	-7.728310	1.541051	-1.211262
C	-6.814380	1.519911	-0.161466
C	-5.437328	1.599846	-0.391865
C	-2.464286	1.465914	-1.612569
C	-1.163974	1.088965	-2.178553
C	-0.175435	1.118918	-1.146696

C	-0.789943	1.555890	0.149569
C	-2.302793	1.475252	-0.111244
C	-0.853667	0.608051	-3.431422
C	0.490678	0.260684	-3.718093
C	1.486636	0.347937	-2.703071
C	1.124536	0.759928	-1.388517
C	2.830872	0.025725	-3.017752
C	3.183747	-0.320489	-4.299975
C	2.213941	-0.386716	-5.330278
C	0.884156	-0.137119	-5.027041
C	-0.148724	-0.214402	-6.075315
N	-0.507419	0.793411	-6.828591
N	-1.482289	0.426199	-7.702448
C	-1.793028	-0.828382	-7.552877
C	-0.960290	-1.469103	-6.441274
C	-1.839716	-1.949711	-5.298658
C	-0.079907	-2.584476	-6.978497
C	2.667010	-0.701744	-6.720169
H	-0.483286	2.597874	0.373054
H	-2.823604	2.346730	0.333696
H	1.883934	0.783813	-0.601916
H	3.585607	0.078279	-2.224794
H	4.228158	-0.538231	-4.548156
H	-2.552083	-1.166234	-5.009425
H	-2.402941	-2.838536	-5.616631
H	-1.213557	-2.208618	-4.435836
H	-2.545827	-1.365079	-8.126654
H	-0.705823	-3.439376	-7.270724
H	0.480827	-2.238212	-7.856242
H	0.623902	-2.902218	-6.198781

H	-1.616819	0.486811	-4.198775
H	-4.753603	1.577633	0.459957
H	-7.179228	1.439583	0.868839
H	-8.800877	1.473628	-0.999951
H	-8.000575	1.662659	-3.362357
H	-6.736678	2.037617	-5.533925
H	-5.071968	2.452935	-7.359107
H	-2.673987	2.742189	-6.818507
H	-1.858785	2.550063	-4.497527
H	3.614218	-0.183314	-6.925047
H	2.825613	-1.786448	-6.817106
H	1.923732	-0.373168	-7.456603
H	-2.719415	0.537014	0.312264
H	-0.477618	0.899536	0.985598

S_0**-opt:** PFCN (M) - ODM2/MRCI (10,11)

C	-2.501091	1.381857	-1.666895
C	-2.304549	1.321658	-0.170930
C	-0.854160	1.775951	0.051136
C	-0.190828	1.433071	-1.249808
C	1.139856	1.214635	-1.482734
C	1.534999	0.734112	-2.762945
C	2.888172	0.376784	-2.990143
C	3.255827	-0.254477	-4.152594
C	2.303830	-0.500579	-5.171038
C	0.999343	-0.056754	-5.009964
C	0.560507	0.523196	-3.782992
C	-0.809408	0.807204	-3.531443
C	-1.170078	1.258353	-2.277432
H	-2.439036	0.279791	0.186327

H	-3.017721	1.989539	0.350725
H	-0.806250	2.870727	0.221959
H	-0.378555	1.253846	0.903903
H	1.890179	1.350654	-0.698689
H	3.626187	0.578605	-2.205709
H	4.289999	-0.579000	-4.307669
C	2.742720	-1.283239	-6.367590
H	1.878767	-1.623719	-6.951186
H	3.304190	-2.166474	-6.031072
H	3.396624	-0.665632	-7.001487
C	0.042441	-0.179216	-6.122025
C	-0.060779	0.825624	-7.283876
C	-1.251315	0.168509	-7.983609
N	-1.634329	-0.899268	-7.346484
N	-0.878346	-1.100421	-6.233878
C	1.157042	0.845196	-8.190960
H	1.346299	-0.157216	-8.595893
H	0.982874	1.537889	-9.026856
H	2.033414	1.182726	-7.623036
C	-0.392996	2.213988	-6.766648
H	-0.504755	2.908812	-7.610801
H	-1.328269	2.181544	-6.201261
H	0.415512	2.565805	-6.113471
H	-1.695795	0.548820	-8.901228
H	-1.559000	0.585235	-4.290562
C	-3.707191	1.515124	-2.274142
C	-5.029930	1.201495	-1.697436
C	-5.403271	0.728186	-0.445288
C	-6.746097	0.413084	-0.218952
C	-7.703237	0.543777	-1.222219

C	-7.343062	0.993913	-2.495362
C	-6.015559	1.319185	-2.720449
C	-5.363987	1.840635	-3.924054
C	-5.905906	2.265453	-5.126130
C	-5.053486	2.878334	-6.049034
C	-3.711032	3.096053	-5.744683
C	-3.153459	2.648408	-4.543576
C	-3.974128	1.972978	-3.648755
H	-4.678159	0.597140	0.361223
H	-7.050788	0.052744	0.770189
H	-8.746853	0.285999	-1.012721
H	-8.086231	1.079766	-3.292571
H	-6.971352	2.145071	-5.338888
H	-5.452249	3.217731	-7.011116
H	-3.083358	3.638345	-6.462329
H	-2.109137	2.857844	-4.298021

S_0**-opt:** PFCN (S) - DFT (B3LYP-BJ/def2-SVP)

C	-2.996802	2.308068	-4.846354
C	-3.861489	2.007275	-3.784098
C	-5.262977	2.045494	-4.021761
C	-5.779670	2.275588	-5.297558
C	-4.896384	2.498154	-6.358138
C	-3.515583	2.533180	-6.126615
C	-3.630806	1.753677	-2.333630
C	-4.983774	1.741068	-1.715868
C	-5.957241	1.870919	-2.745693
C	-7.322496	1.824723	-2.466258
C	-7.741174	1.648731	-1.142991
C	-6.795135	1.509435	-0.121472

C	-5.422438	1.546757	-0.398109
C	-2.458070	1.511871	-1.667281
C	-1.137878	1.136048	-2.195070
C	-0.158397	1.201034	-1.153642
C	-0.795165	1.701049	0.116629
C	-2.310322	1.546194	-0.149750
C	-0.779302	0.638521	-3.435492
C	0.557106	0.262121	-3.714898
C	1.537379	0.356929	-2.671118
C	1.141031	0.818682	-1.383333
C	2.877021	-0.003849	-2.967362
C	3.233249	-0.393278	-4.237654
C	2.284726	-0.466833	-5.297674
C	0.952767	-0.170837	-5.026445
C	-0.084993	-0.233286	-6.091753
N	-0.389366	0.791402	-6.812294
N	-1.421428	0.423803	-7.742821
C	-1.715461	-0.809863	-7.557589
C	-0.917456	-1.460411	-6.457168
C	-1.824674	-1.941489	-5.304277
C	-0.055733	-2.629918	-6.971922
C	2.775809	-0.830306	-6.677110
H	-0.542181	2.764972	0.272929
H	-2.898707	2.354255	0.304493
H	1.888479	0.865456	-0.586053
H	3.629080	0.055832	-2.176055
H	4.276901	-0.635627	-4.456839
H	-2.538980	-1.166048	-4.994999
H	-2.398647	-2.824297	-5.626292
H	-1.215418	-2.223717	-4.432201

H	-2.487480	-1.307010	-8.154203
H	-0.702985	-3.465152	-7.281032
H	0.560361	-2.333580	-7.832005
H	0.608994	-2.987886	-6.171247
H	-1.523810	0.531021	-4.213683
H	-4.724380	1.420450	0.425460
H	-7.127793	1.367473	0.909799
H	-8.807806	1.615303	-0.907312
H	-8.056299	1.922038	-3.270446
H	-6.860090	2.293749	-5.462138
H	-5.283159	2.665755	-7.366421
H	-2.825115	2.718500	-6.951364
H	-1.921170	2.383882	-4.707386
H	3.764997	-0.383180	-6.860984
H	2.889767	-1.921336	-6.792734
H	2.094974	-0.473058	-7.460537
H	-2.673781	0.596744	0.283922
H	-0.453567	1.156723	1.010361

S_0**-opt:** PFCN (M) - DFT (B3LYP-BJ/def2-SVP)

C	-2.573770	1.509107	-1.649456
C	-2.364546	1.492159	-0.140025
C	-0.882922	1.882158	0.058021
C	-0.242826	1.445073	-1.232714
C	1.068574	1.129014	-1.481659
C	1.454136	0.618643	-2.752693
C	2.782627	0.186009	-3.001314
C	3.111286	-0.418589	-4.190545
C	2.158849	-0.575567	-5.236362
C	0.869156	-0.083886	-5.058206

C	0.466694	0.467243	-3.785889
C	-0.884074	0.807677	-3.498178
C	-1.242547	1.291639	-2.246712
H	-2.535279	0.472945	0.253870
H	-3.054415	2.166602	0.383731
H	-0.792959	2.977081	0.178050
H	-0.423896	1.419412	0.944418
H	1.825298	1.216964	-0.696735
H	3.531785	0.304429	-2.213917
H	4.125096	-0.798094	-4.346537
C	2.582784	-1.331139	-6.471428
H	1.728582	-1.601943	-7.103841
H	3.086524	-2.266279	-6.177981
H	3.301005	-0.758967	-7.081240
C	-0.107632	-0.057082	-6.177397
C	-0.100256	0.990035	-7.293677
C	-1.400764	0.572138	-7.930413
N	-1.955709	-0.430470	-7.356329
N	-1.152299	-0.810692	-6.229048
C	1.064587	0.878019	-8.296459
H	1.126675	-0.123096	-8.744151
H	0.922282	1.609567	-9.107315
H	2.019099	1.104836	-7.800504
C	-0.138579	2.416146	-6.710916
H	-0.239958	3.156242	-7.519795
H	-0.976149	2.543945	-6.016895
H	0.794459	2.622559	-6.164422
H	-1.843743	1.035730	-8.818296
H	-1.644841	0.592458	-4.244170
C	-3.795804	1.670015	-2.244629

C	-5.114951	1.472197	-1.590799
C	-5.476546	1.086729	-0.292518
C	-6.828030	0.899375	0.022802
C	-7.825580	1.075248	-0.943274
C	-7.481639	1.437171	-2.250010
C	-6.138199	1.632830	-2.567581
C	-5.512145	2.008578	-3.836438
C	-6.084469	2.359335	-5.060353
C	-5.254289	2.789950	-6.099563
C	-3.872194	2.900512	-5.896427
C	-3.297395	2.541278	-4.673482
C	-4.105355	2.047643	-3.645254
H	-4.731553	0.919328	0.482127
H	-7.104048	0.607512	1.039262
H	-8.874437	0.921393	-0.677681
H	-8.255200	1.559655	-3.012357
H	-7.167470	2.315324	-5.199850
H	-5.686271	3.062876	-7.065449
H	-3.234794	3.276185	-6.700708
H	-2.228812	2.670329	-4.515661

S_0**-opt:** PFCN (S) - MRSF TD-DFT (CAM-B3LYP/cc-pVDZ)

C	-3.0554584015	2.3182380794	-4.8393438220
C	-3.9071626242	2.0110741478	-3.7696634719
C	-5.3018840053	2.1228439494	-3.9810389473
C	-5.8314028863	2.4232507479	-5.2303321100
C	-4.9656452087	2.6429198740	-6.3000325471
C	-3.5844138209	2.6078029014	-6.0940890715
C	-3.6525145345	1.7209730392	-2.3295187268
C	-4.9957643928	1.7485712455	-1.6970369602

C	-5.9792179588	1.9483021592	-2.7001509033
C	-7.3351474627	1.9589182095	-2.4054367397
C	-7.7473938779	1.7697763216	-1.0868948063
C	-6.7959451322	1.5562138413	-0.0881334404
C	-5.4346234804	1.5343455070	-0.3817795447
C	-2.4982911368	1.4556152628	-1.6981140545
C	-1.1728030074	1.1307702293	-2.2231722440
C	-0.2046988388	1.1607213984	-1.1757966326
C	-0.8560689400	1.6036431549	0.1036380812
C	-2.3578946287	1.4165090075	-0.1743093498
C	-0.7779806722	0.6386261917	-3.4813295898
C	0.5497507353	0.2834585436	-3.7427942907
C	1.5258846822	0.3934804417	-2.6936949906
C	1.0969012500	0.8181773470	-1.3958668407
C	2.8619800942	0.0767416406	-2.9718198378
C	3.2509096163	-0.2973996779	-4.2430787467
C	2.3176315745	-0.4112393820	-5.3015211984
C	0.9774387902	-0.1689240455	-5.0457897649
C	-0.0472506684	-0.3012960374	-6.1188816431
N	-0.3984799372	0.7026346039	-6.8392756800
N	-1.4054622846	0.2811686370	-7.7726726306
C	-1.6315434387	-0.9611521685	-7.5804548376
C	-0.8080735108	-1.5684262760	-6.4830898576
C	-1.6878636789	-2.0943883715	-5.3368610238
C	0.0986890095	-2.6938324153	-7.0025677350
C	2.8392258305	-0.7638447365	-6.6714489358
H	-0.6341132319	2.6670446087	0.2911017434
H	-2.9561350697	2.1978970879	0.3092891016
H	1.8317030517	0.8598790300	-0.5885733916
H	3.6019910573	0.1536912070	-2.1724513450

H	4.3033122467	-0.5017431587	-4.4493046710
H	-2.4349659769	-1.3552033366	-5.0184275999
H	-2.2217493206	-2.9999986700	-5.6617611806
H	-1.0677189716	-2.3560053171	-4.4672976881
H	-2.3734291967	-1.4957632064	-8.1798079562
H	-0.5146330594	-3.5528874047	-7.3127264524
H	0.6986694861	-2.3714706047	-7.8637103065
H	0.7792824220	-3.0326846198	-6.2077694670
H	-1.5138382709	0.5179662162	-4.2662400224
H	-4.7392593667	1.3428219434	0.4290920891
H	-7.1193259871	1.3977994699	0.9421062222
H	-8.8095964785	1.7806678876	-0.8379615758
H	-8.0712809015	2.1109534696	-3.1970311166
H	-6.9115671724	2.5004729851	-5.3685725740
H	-5.3642368310	2.8679331117	-7.2904617140
H	-2.9020120521	2.7991037777	-6.9229122665
H	-1.9765523180	2.3581459061	-4.7171398730
H	3.8097789675	-0.2758831211	-6.8407977833
H	2.9991355996	-1.8482636444	-6.7793719535
H	2.1584496628	-0.4361283391	-7.4663336452
H	-2.7017284527	0.4463663076	0.2255134617
H	-0.5037882375	1.0380169068	0.9773397190

S_1**-opt** PFCN - ODM2/MRCI (10,11)

C	-3.749069	3.702704	-3.649641
C	-4.060601	2.377065	-3.337744
C	-5.039727	1.652216	-4.096577
C	-5.705384	2.277955	-5.147593
C	-5.388788	3.596223	-5.448953
C	-4.423516	4.294667	-4.710211

C	-3.578676	1.538102	-2.263569
C	-4.166910	0.231027	-2.476037
C	-5.103290	0.311909	-3.563646
C	-5.843598	-0.812313	-3.919299
C	-5.636681	-2.001704	-3.229491
C	-4.712869	-2.075479	-2.178727
C	-3.968550	-0.968682	-1.786168
C	-2.355292	1.665341	-1.604625
C	-1.085359	1.246080	-2.145076
C	-0.074905	1.312467	-1.136129
C	-0.653108	1.877495	0.132848
C	-2.140192	2.109152	-0.185737
C	-0.842246	0.760918	-3.419594
C	0.441788	0.242659	-3.690710
C	1.464366	0.321093	-2.692778
C	1.193650	0.873184	-1.404465
C	2.762102	-0.154140	-3.004615
C	3.037644	-0.664513	-4.252233
C	2.031732	-0.773632	-5.243550
C	0.742239	-0.350315	-4.949893
C	-0.325796	-0.535976	-5.947679
N	-0.410324	0.172000	-7.043867
N	-1.428918	-0.266415	-7.829140
C	-2.018733	-1.294031	-7.290818
C	-1.388327	-1.651068	-5.944126
C	-2.417146	-1.559159	-4.829465
C	-0.724280	-3.019002	-5.976318
C	2.379696	-1.331788	-6.585917
H	-0.139835	2.821545	0.393015
H	-2.421818	3.172472	-0.114427

H	1.992560	0.927653	-0.662204
H	3.544768	-0.093424	-2.242526
H	4.052656	-0.998017	-4.497533
H	-2.963250	-0.609839	-4.877379
H	-3.143306	-2.375839	-4.933075
H	-1.924967	-1.651692	-3.852218
H	-2.870375	-1.825033	-7.710864
H	-1.492155	-3.797973	-6.072187
H	-0.035073	-3.092082	-6.829176
H	-0.166636	-3.177423	-5.043689
H	-1.621431	0.802601	-4.182888
H	-3.258789	-1.037656	-0.956357
H	-4.579367	-3.029273	-1.651697
H	-6.210943	-2.894402	-3.497924
H	-6.575720	-0.754378	-4.733110
H	-6.464130	1.734216	-5.722498
H	-5.897833	4.103914	-6.274377
H	-4.197658	5.334552	-4.979016
H	-3.000881	4.259653	-3.077930
H	3.467621	-1.460426	-6.679152
H	1.897796	-2.311315	-6.717558
H	2.030988	-0.649838	-7.367225
H	-2.803223	1.524978	0.471924
H	-0.528563	1.153995	0.960031

S_1**-opt** PFCN - MRSF TD-DFT (CAM-B3LYP/cc-pVDZ)

C	2.128119	0.181064	-2.435003
C	2.418476	1.063423	-1.230201
N	2.219907	2.308039	-1.470286
N	1.749634	2.455108	-2.816814

C	1.706531	1.294594	-3.348955
C	2.993280	0.626659	0.073813
C	2.119975	0.071844	1.082683
C	2.673334	-0.417342	2.325108
C	4.060219	-0.304516	2.521952
C	4.855548	0.275522	1.563637
C	4.344114	0.756561	0.325730
C	1.830860	-0.992263	3.336785
C	0.493433	-1.076359	3.133832
C	-0.072629	-0.574552	1.902355
C	0.743627	0.023285	0.902406
C	-0.614939	-1.651946	3.974986
C	-1.854238	-1.540959	3.068745
C	-1.416454	-0.846267	1.813327
C	-2.283920	-0.572852	0.681443
C	-3.083997	0.572971	0.477346
C	-3.808380	0.411635	-0.764862
C	-3.433401	-0.858961	-1.316141
C	-2.489945	-1.460751	-0.396599
C	-4.678993	1.412112	-1.200979
C	-4.853195	2.565012	-0.446057
C	-4.147746	2.728206	0.766357
C	-3.278442	1.762057	1.230303
C	-3.790776	-1.531842	-2.486057
C	-3.249773	-2.780486	-2.770959
C	-2.332559	-3.373264	-1.875655
C	-1.950954	-2.738217	-0.710336
C	5.316374	1.374256	-0.643806
C	1.006412	-0.853771	-2.255734
C	3.406253	-0.511655	-2.940508

H	-0.733048	-1.074628	4.904803
H	-2.696088	-0.976561	3.507896
H	2.298287	-1.361884	4.251493
H	4.496444	-0.666138	3.454208
H	5.926694	0.377020	1.750299
H	0.046970	-0.400637	-1.973640
H	0.846926	-1.391081	-3.201876
H	1.275735	-1.600641	-1.495163
H	1.370592	1.162637	-4.380637
H	3.192640	-1.054076	-3.872793
H	4.211987	0.208562	-3.138157
H	3.761603	-1.243013	-2.199072
H	0.258564	0.429352	0.018576
H	-1.238266	-3.223693	-0.037691
H	-1.919287	-4.357400	-2.109536
H	-3.532955	-3.306314	-3.683794
H	-4.501306	-1.074541	-3.179744
H	-5.224585	1.286107	-2.139896
H	-5.532718	3.346872	-0.787604
H	-4.292694	3.641291	1.348215
H	-2.742456	1.921643	2.169681
H	6.064318	1.969784	-0.101201
H	5.862995	0.597064	-1.201255
H	4.816641	2.033324	-1.361241
H	-2.297333	-2.514266	2.790787
H	-0.398136	-2.688443	4.271367

MECI$_{in}$ of PFCN - ODM2/MRCI (10,11)

C	-3.190815	4.191922	-3.751531
C	-3.730354	2.953219	-3.395755

C	-4.496654	2.212919	-4.349224
C	-4.726364	2.720532	-5.627470
C	-4.165598	3.941723	-5.968022
C	-3.406942	4.666898	-5.037943
C	-3.889944	2.363344	-2.021071
C	-4.396265	1.005802	-2.378926
C	-4.922597	0.991751	-3.708256
C	-5.725977	-0.062455	-4.145653
C	-5.952013	-1.131200	-3.284416
C	-5.411163	-1.135186	-1.991461
C	-4.637726	-0.077359	-1.526875
C	-2.555680	2.294262	-1.550453
C	-1.341002	1.689371	-2.103999
C	-0.234070	1.928386	-1.233059
C	-0.671213	2.701404	-0.020459
C	-2.148024	3.010543	-0.294537
C	-1.202175	0.998341	-3.295436
C	0.078456	0.500531	-3.634801
C	1.192941	0.773344	-2.780839
C	1.021848	1.487826	-1.561611
C	2.490294	0.357681	-3.178975
C	2.681578	-0.256856	-4.392956
C	1.589834	-0.553481	-5.246428
C	0.302666	-0.222746	-4.843358
C	-0.834007	-0.642142	-5.681685
N	-1.154157	-0.067251	-6.810952
N	-2.192230	-0.710770	-7.405387
C	-2.567786	-1.736580	-6.698258
C	-1.723033	-1.874213	-5.430933
C	-2.581677	-1.771343	-4.182071

C	-0.908250	-3.159305	-5.445469
C	1.852104	-1.196854	-6.571060
H	-0.067567	3.620335	0.089834
H	-2.319198	4.081805	-0.472397
H	1.885035	1.682764	-0.922243
H	3.336278	0.560149	-2.515637
H	3.692115	-0.528893	-4.719398
H	-3.229437	-0.890525	-4.229216
H	-3.216225	-2.663941	-4.099135
H	-1.940744	-1.711722	-3.291557
H	-3.384410	-2.410894	-6.948680
H	-1.581614	-4.021660	-5.345744
H	-0.351490	-3.252522	-6.388205
H	-0.201183	-3.158424	-4.605005
H	-2.051079	0.885263	-3.973252
H	-4.243977	-0.086199	-0.505030
H	-5.611149	-1.990325	-1.332607
H	-6.572342	-1.973611	-3.608575
H	-6.176597	-0.036595	-5.144323
H	-5.334793	2.157720	-6.344130
H	-4.318536	4.353606	-6.970558
H	-2.980677	5.634160	-5.336447
H	-2.623796	4.791340	-3.029943
H	2.932670	-1.235058	-6.771838
H	1.459266	-2.223231	-6.576204
H	1.363499	-0.617972	-7.360901
H	-2.810137	2.684860	0.518921
H	-0.553351	2.077375	0.885141

MECI$_{out}$ of PFCN - ODM2/MRCI (10,11)

C	-3.478092	4.462778	-4.112617
C	-4.056567	3.342835	-3.522912
C	-5.458417	3.315621	-3.273510
C	-6.270119	4.388824	-3.638864
C	-5.676529	5.500947	-4.213926
C	-4.295858	5.537306	-4.445487
C	-3.434813	2.000568	-3.296642
C	-4.528452	1.338906	-2.489884
C	-5.759301	2.051638	-2.638839
C	-6.965011	1.480260	-2.236003
C	-6.943839	0.220438	-1.642091
C	-5.737886	-0.475485	-1.485955
C	-4.528135	0.064264	-1.909433
C	-2.383888	2.054905	-2.348179
C	-1.140322	1.343894	-2.595533
C	-0.275478	1.407038	-1.463765
C	-0.919930	2.207021	-0.365734
C	-2.273725	2.635576	-0.952354
C	-0.811647	0.668953	-3.756538
C	0.428449	-0.010154	-3.794538
C	1.313746	0.072936	-2.674190
C	0.949214	0.791492	-1.498714
C	2.582704	-0.559294	-2.751169
C	2.968777	-1.210074	-3.897446
C	2.102743	-1.311630	-5.015888
C	0.835726	-0.750875	-4.943110
C	-0.102834	-0.912950	-6.068815
N	-0.044440	-0.197542	-7.161365
N	-0.990768	-0.592720	-8.052304
C	-1.686032	-1.585898	-7.579974

C	-1.212140	-1.975403	-6.178731
C	-2.325676	-1.817095	-5.155925
C	-0.631894	-3.382184	-6.164511
C	2.587067	-2.023536	-6.239072
H	-0.290825	3.080114	-0.111599
H	-2.350270	3.729308	-1.052773
H	1.643291	0.839509	-0.657504
H	3.254158	-0.502114	-1.889211
H	3.964042	-1.664482	-3.963452
H	-2.788663	-0.827290	-5.231633
H	-3.097222	-2.578249	-5.336678
H	-1.923396	-1.956198	-4.142960
H	-2.509590	-2.079321	-8.092073
H	-1.435934	-4.112491	-6.329350
H	0.118461	-3.496600	-6.957930
H	-0.164355	-3.579074	-5.190241
H	-1.498841	0.695098	-4.603981
H	-3.600620	-0.507502	-1.799066
H	-5.752076	-1.469388	-1.019131
H	-7.877426	-0.240213	-1.303216
H	-7.909103	2.015093	-2.388670
H	-7.353981	4.345669	-3.478046
H	-6.289896	6.361450	-4.503020
H	-3.856000	6.429675	-4.908834
H	-2.405872	4.494510	-4.328799
H	2.270337	-3.076124	-6.200421
H	2.183077	-1.551991	-7.140308
H	3.684928	-1.987975	-6.285614
H	-3.117332	2.278498	-0.348156
H	-1.050568	1.577468	0.534343

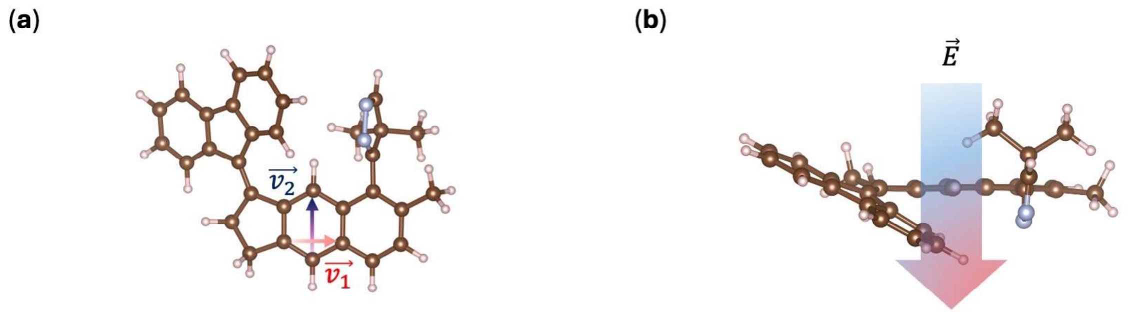

Figure S1: **Vectors and orientation of electric field.** (a) Definition of vectors spanning ’stator plane’ - $\vec{v}_1, \vec{v}_2$; (b) orientation of electric field vector, $\vec{E}$.

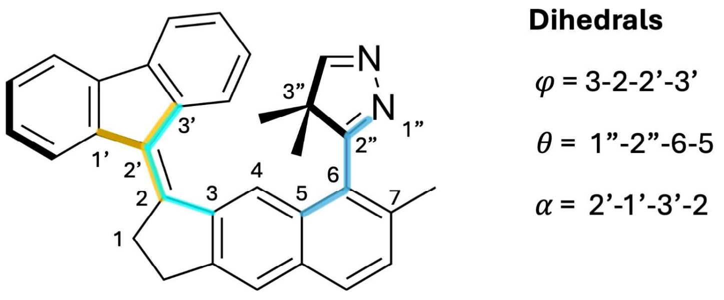

PFCN

Figure S2: **Definition of key coordinates used to characterize the PFCN structure.**

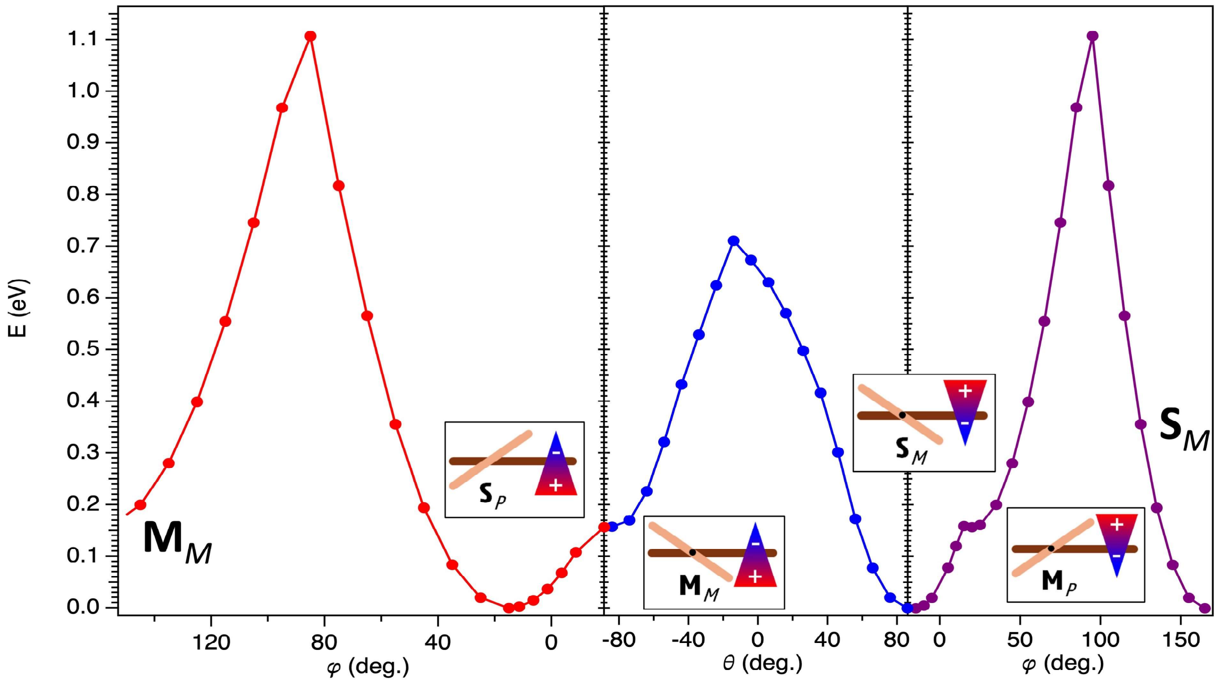

Figure S3: **Relaxed scans optimized in the S_0 state for PFCN.** Energy profiles along the switch rotation (about the θ dihedral angle, central panel), and along rotor rotation (about the φ dihedral angle, side panels), obtained at the B3LYP-BJ/def2-SVP level of theory.

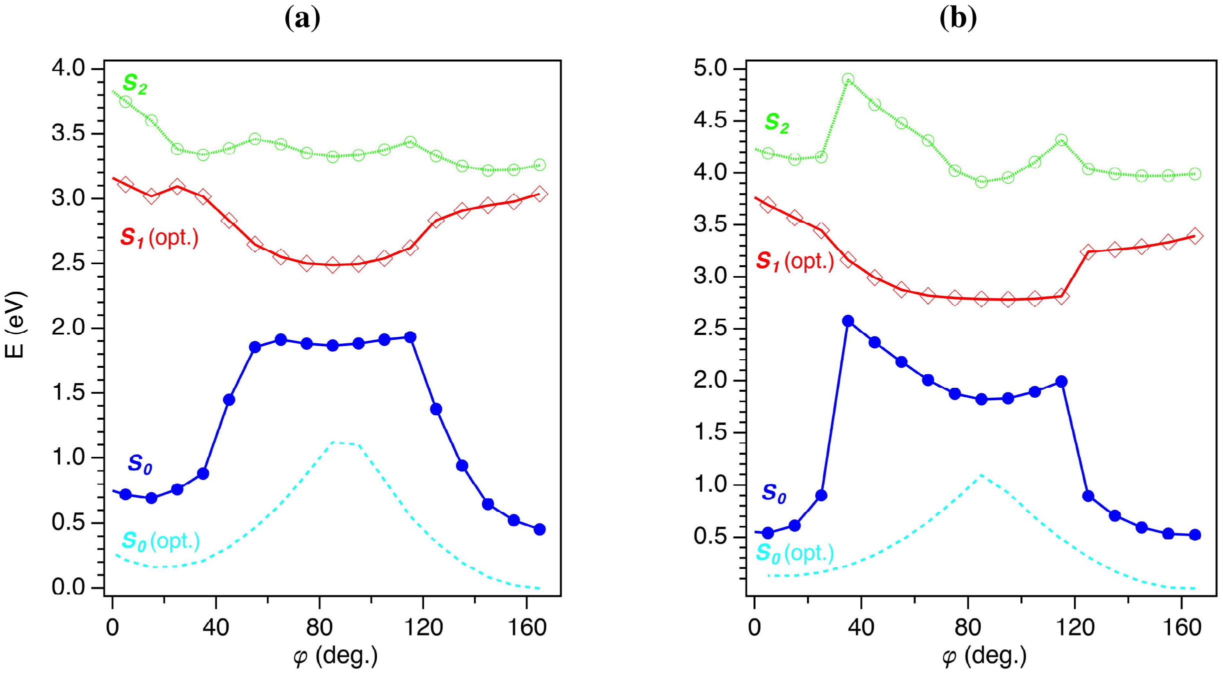

Figure S4: **Relaxed scans along the C–C dihedral (φ) optimized in the S_1 state for PFCN.** Graphs represent energy profiles obtained using **(a)** MRSF, and **(b)** ODM2/MRCI methods.

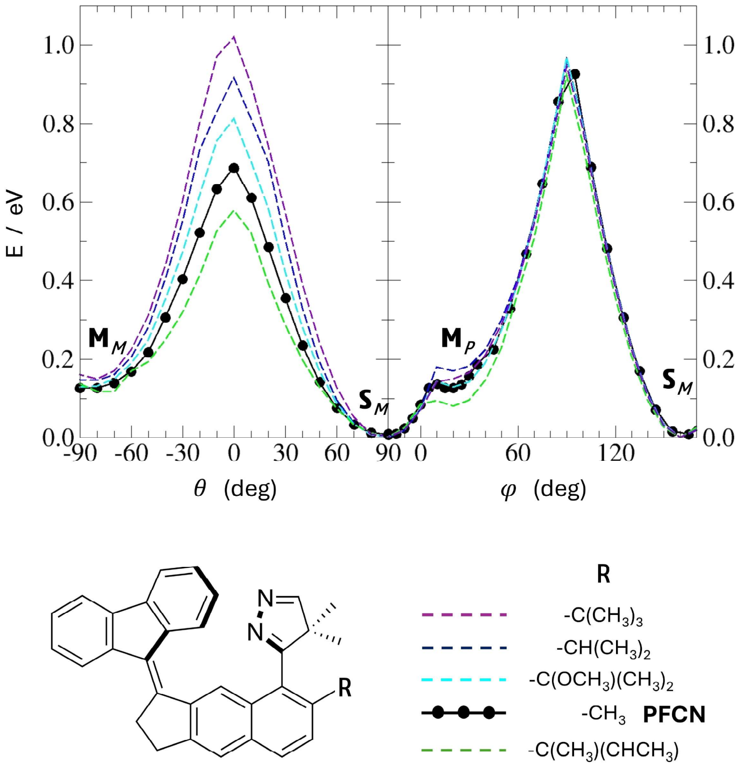

Figure S5: **Relaxed scans of 7-substituted PFCN derivatives.** Ground-state potential energy profiles calculated along the rotor rotation coordinate (left panel), and for the PFCN chirality switching (right panel), obtained at the ODM2/MRCI level of theory.

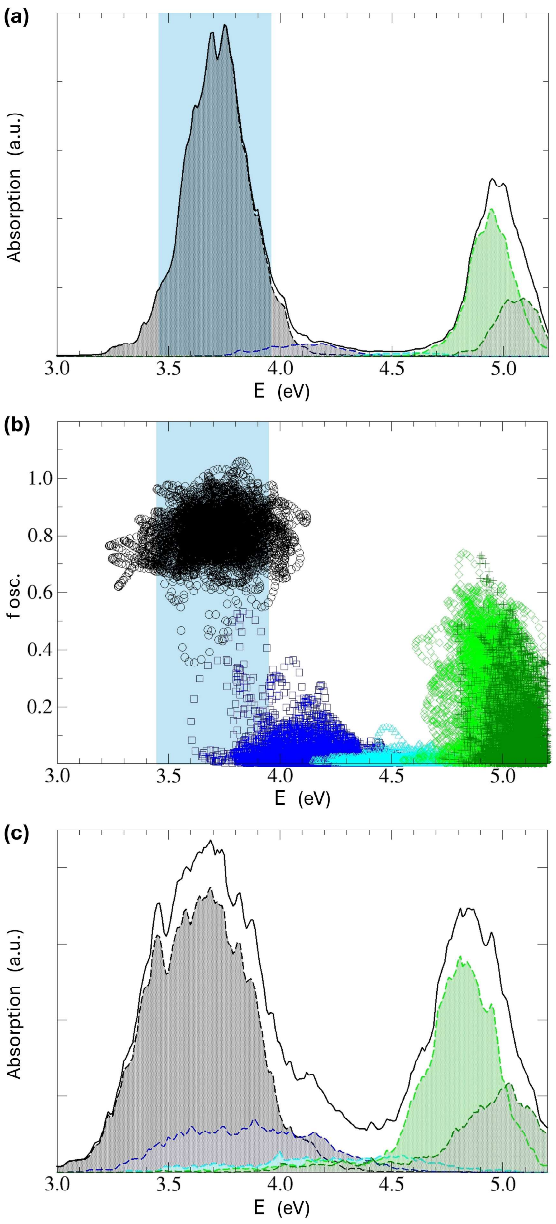

Figure S6: **Dynamically-averaged absorption properties of the PFCN system.** UV-Vis absorption spectrum obtained from MD at 298 K **(a)**, and respective transition-energy vs. oscillator-strength correlation plot **(b)**, the UV-Vis absorption spectrum obtained from MD at 900 K **(c)**. The blue-shaded areas mark energy window employed in NAMD initial conditions generation. The dashed lines (panels (a), (c)) and the symbols (panel (b)) of a given color mark respective data for different excited states: S_1 – black (circles), S_2 – blue (squares), S_3 – cyan (triangles), S_4 – light green (diamonds), and S_5 – dark green (plus signs). The black solid line in panels (a), (c) marks the total simulated absorption spectrum of PFCN.

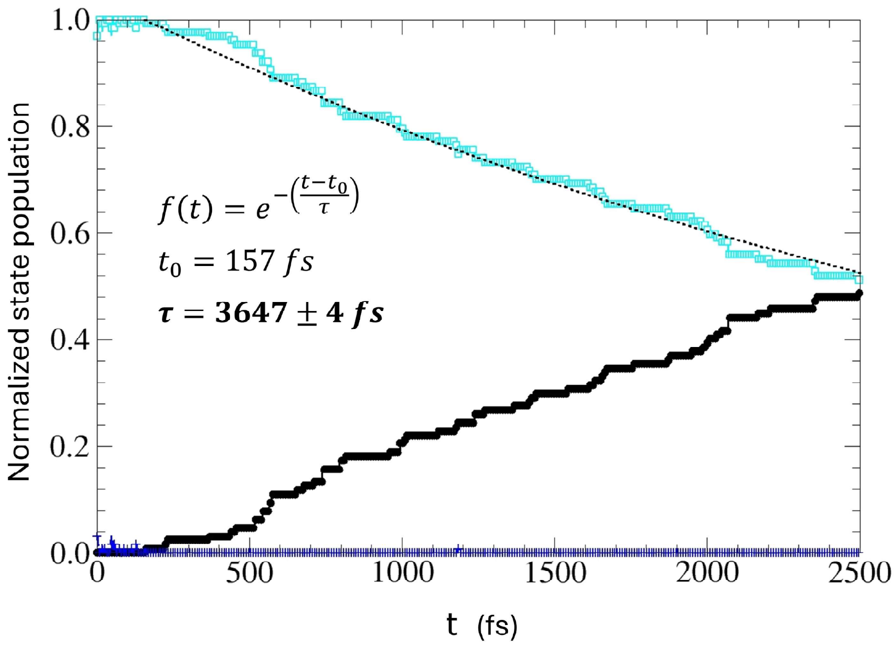

Figure S7: **Normalized mean electronic-state populations in NAMD simulations of the PFCN S-form photorelaxation**. Black full circles – S_0, empty cyan squares – S_1, blue crosses – S_2, black dotted line – exponential fit to the mean S_1 population decay, with the fitted function and its parameters given as inset.

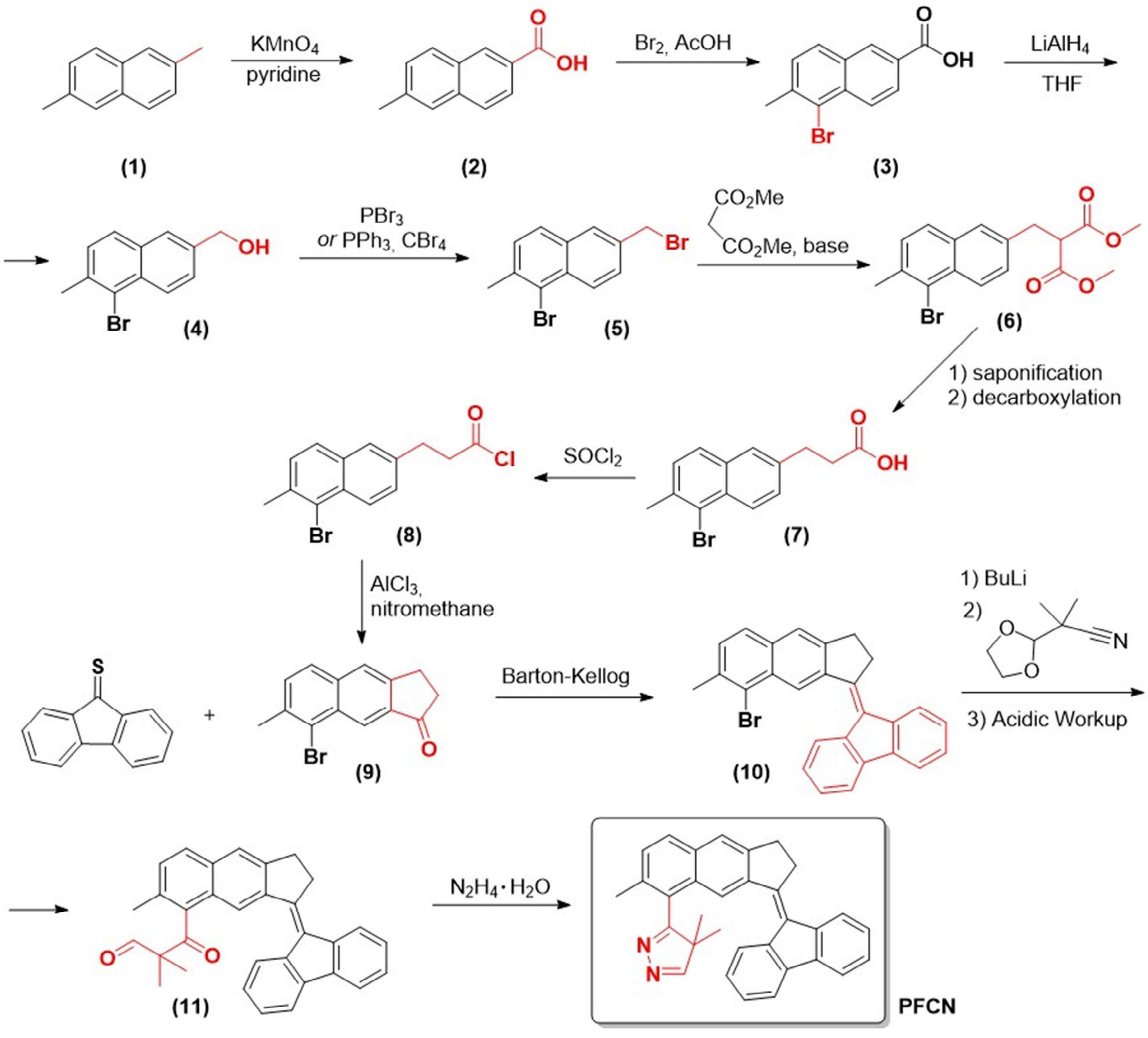

Figure S8: **The outline of the possible synthesis path for the PFCN system.**

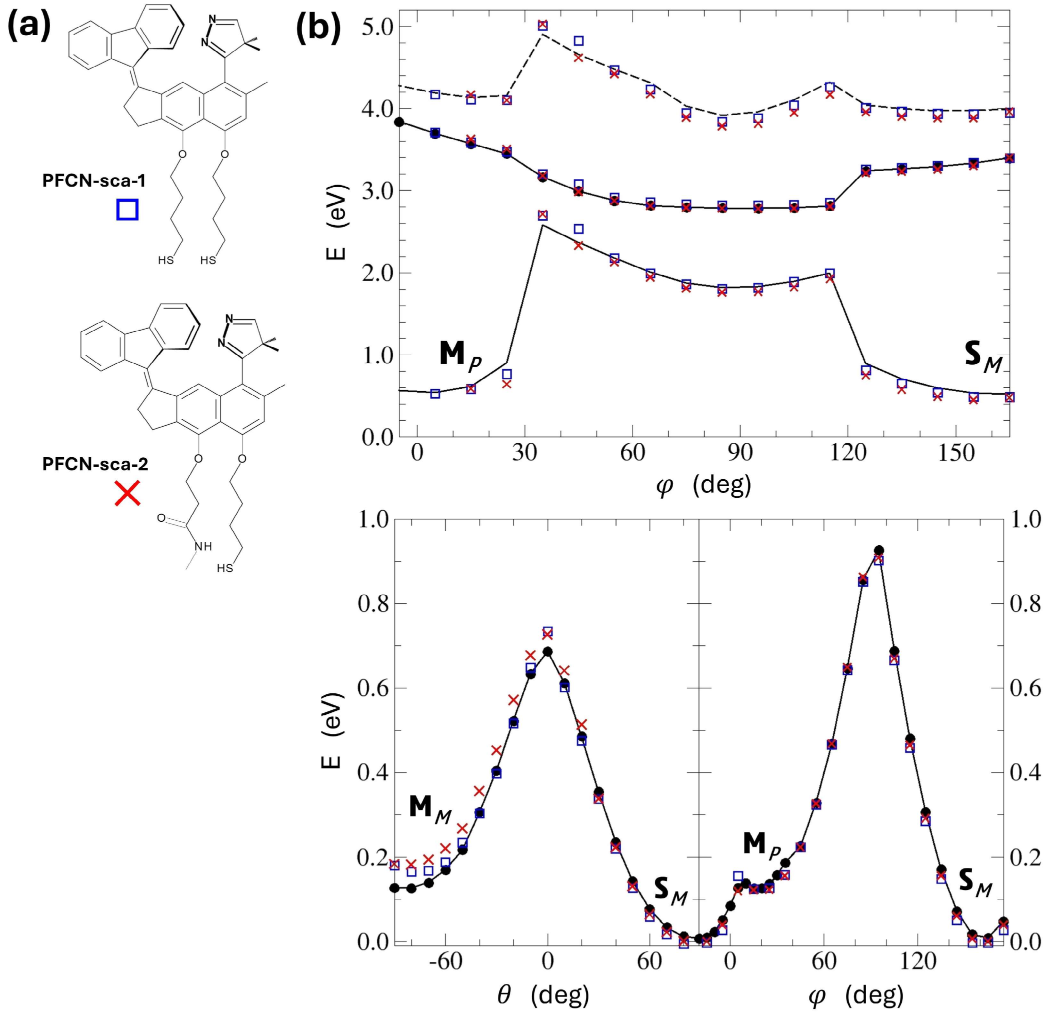

Figure S9: **Linkers' impact on the PES landscape of PFCN.** (a) Chemical structures of the two designed PFCN derivatives including linkers facilitating system deposition on a surface. (b) Potential energy profile scans determined at the ODM2/MRCI level of theory for: the bare PFCN molecule (black lines, reproduced from the main text), PFCN-sca-1 (blue empty squares), and PFCN-sca-2 (red crosses). Upper panel: profiles optimized in the lowest excited electronic state (illustrating the optical step of the motor operation). Lower panel: optimized ground-state potential energy profiles for the thermal motor step (right), and for the motor chirality switching (left).

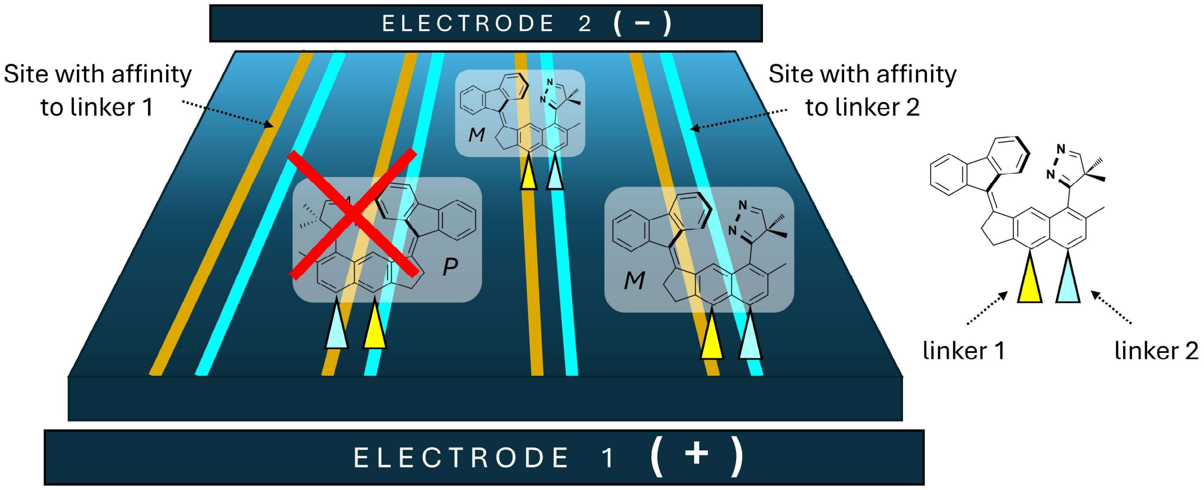

Figure S10: **Schematic illustration of the proposed strategy for parallel alignment of the E-motor molecules on a pre-structured surface.** The orange / turquoise thick lines correspond to sites capable of binding linker 1 (yellow triangle) / linker 2 (light-blue triangle), respectively. Dark-blue color marks areas inactive for the linkers binding (e.g., covered with an inert masking layer).

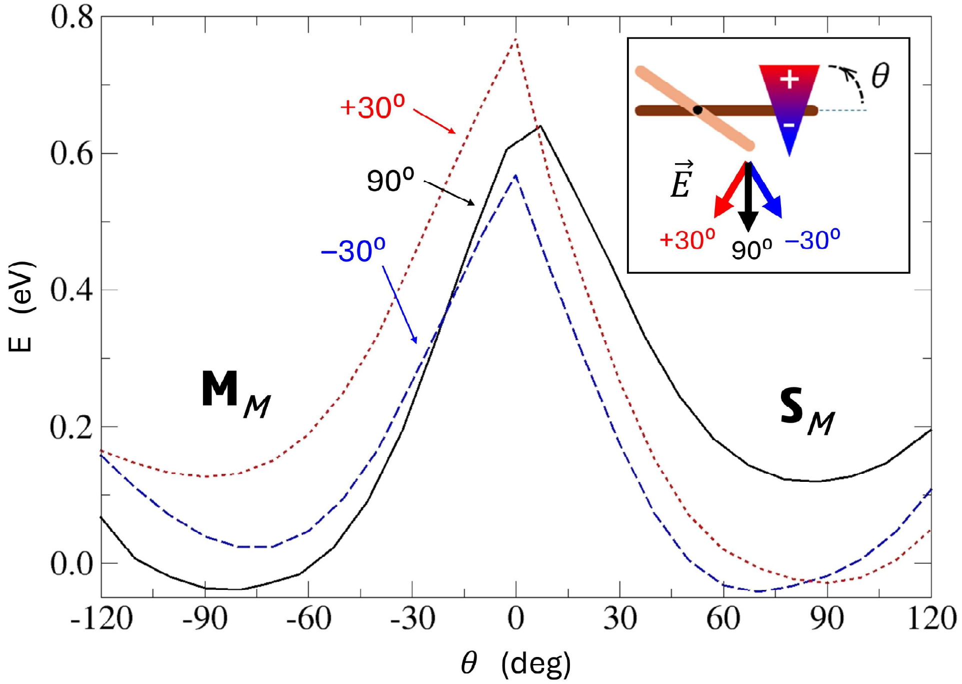

Figure S11: **Impact of the imperfect alignment of the E-motor molecules on the efficiency of their switching.** Potential energy profile scans calculated at the B3LYP-BJ/def2-SVP level of theory for the PFCN system along the switching-unit rotation coordinate under the external electric field of 0.004 a.u. oriented: perpendicularly to the stator plane (90°, solid black line), rotated counterclockwise by 30° (–30°, blue dashed line), rotated clockwise by 30° (+30°, red dotted line).

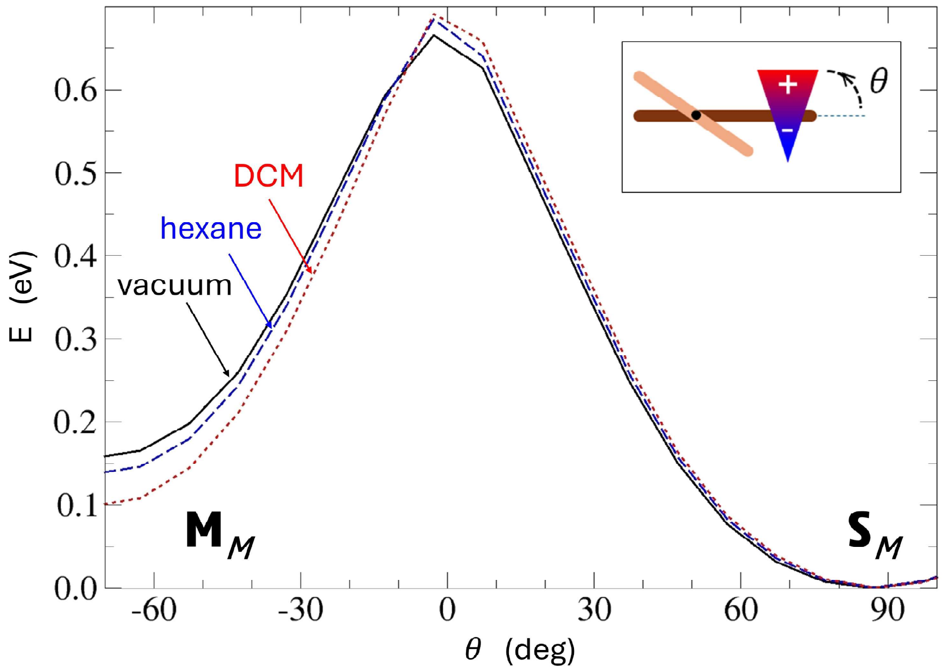

Figure S12: **Impact of solvent on the PFCN switching barrier in the absence of the field.** Potential energy profile scans calculated at the B3LYP-BJ/def2-SVP level of theory for the PFCN system along the switching-unit rotation coordinate: in vacuum (solid black line), in hexane (blue dashed line), and in DCM (red dotted line). The solvent environment has been modeled with COSMO, employing the TURBOMOLE software.

Table S1: **Active space molecular orbitals included in the calculations at the ODM2/MRCI-SD level of theory for S form of PFCN.** 10 electrons distributed in 11 orbitals, on the example of the **S**$_M$ structure.

HOMO-4	HOMO-3	HOMO-2	HOMO-1
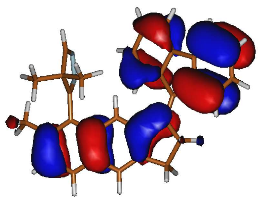	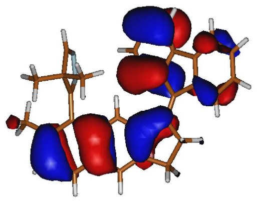	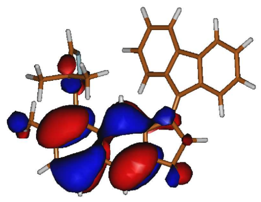	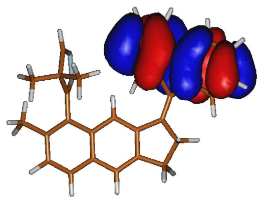
HOMO	LUMO	LUMO+1	LUMO+2
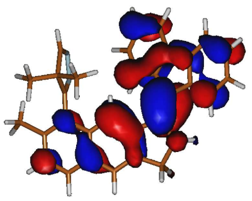	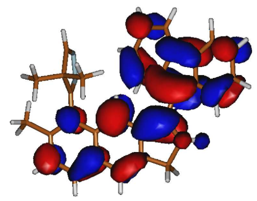	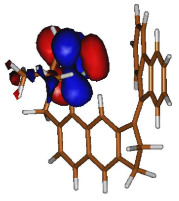	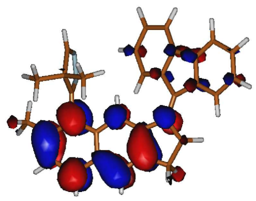
LUMO+3	LUMO+4	LUMO+5	
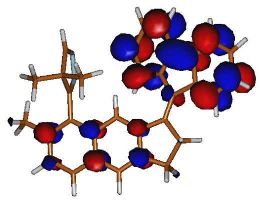	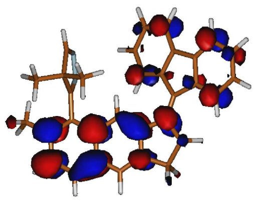	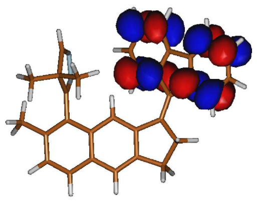	

Table S2: **Active space molecular orbitals included in the calculations at the ODM2/MRCI-SD level of theory for M form of PFCN.** 10 electrons distributed in 11 orbitals, on the example of the $\mathbf{M}_M$ structure.

HOMO-4	HOMO-3	HOMO-2	HOMO-1
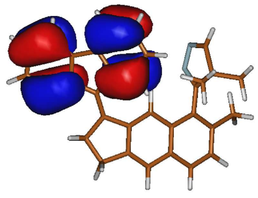	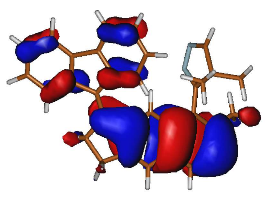	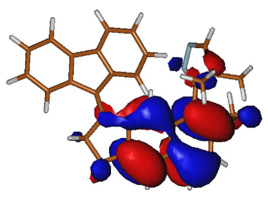	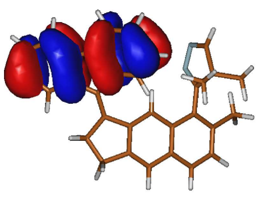
HOMO	LUMO	LUMO+1	LUMO+2
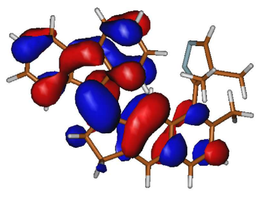	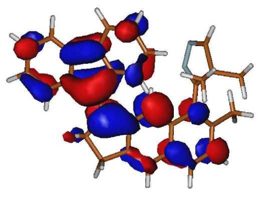	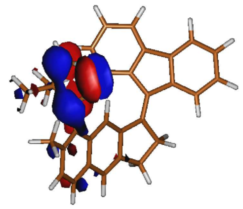	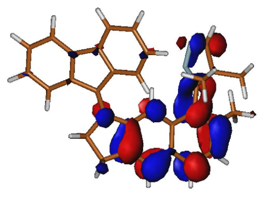
LUMO+3	LUMO+4	LUMO+5	
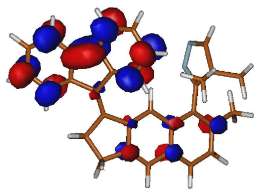	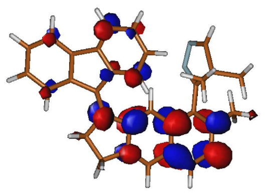	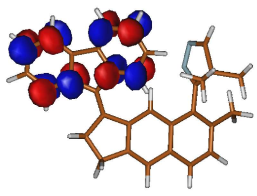	

Table S3: **Comparison of relative energies (eV, [kcal/mol]) and characteristic dihedral angles (deg.) of the S and M forms.** Structures are optimized at the ODM2/MRCI and B3LYP-BJ/def2-SVP level of theory, on the example of the $\mathbf{S}_M$ and $\mathbf{M}_P$ structures.

	**ODM2/MRCI**		**DFT**	
	**S**	**M**	**S**	**M**
E_{rel}	-	0.171 [4.0]	-	0.156 [3.6]
θ	88	-79	86	-72
φ	-13	-20	-15	-19
α	-3	-1	-1	0

Table S4: **Comparison of the ground-state energy barriers (eV) determined at the ODM2/MRCI and at the B3LYP-BJ/def2-SVP levels of theory.** It includes all studied **S↔M** transformations: **S→M (thermal)** – S-to-M transformation by positive φ rotation, **M→S (thermal)** – M-to-S transformation by negative φ rotation, **S→M (optical)** – S-to-M transformation by negative φ rotation, **M→S (optical)** – M-to-S transformation by positive φ rotation, **S→M (switch)** – S-to-M transformation by positive θ rotation, and **M→S (switch)** – M-to-S transformation by negative θ rotation.

	**ODM2/MRCI**	**B3LYP-BJ/def2-SVP**
S→M (thermal)	0.130	0.159
M→S (thermal)	0.011	—
S→M (optical)	0.918	1.107
M→S (optical)	0.798	0.946
S→M (switch)	0.677	0.710
M→S (switch)	0.560	0.553

Table S5: **Absorption properties of S and M forms of PFCN calculated at the ODM2/MRCI level.** Calculations are performed on the example of the $\mathbf{S}_M$ and $\mathbf{M}_P$ structures. E_{ex} is excitation energy of the $S_0 \rightarrow S_i$ transition (eV), f is the corresponding oscillator strength, and El. config. is the leading electronic configuration change.

**Transition**	E_{ex}	f	**El. config.**
**S**			
$S_0 \rightarrow S_1$	3.77	0.4641	H → L (91%)
$S_0 \rightarrow S_2$	4.17	0.0052	H-1 → L (85%)
**M**			
$S_0 \rightarrow S_1$	3.77	0.2611	H → L (91%)
$S_0 \rightarrow S_2$	4.20	0.0034	H-1 → L (84%)

Table S6: **Properties of the $S_0 \rightarrow S_1$ transition determined at different levels of theory for the isolated PFCN and 4a-H systems**, calculated at their ground-state structures optimized at the ODM2/MRCI level of theory. Excitation energies are given in eV, oscillator strengths are shown in parentheses. The reference experimental value for the **4a**-H system (measured in hexane) has been taken from Ref. (*41*). In the ODM2/MRCI calculations, the active spaces including 10 electrons in 11 orbitals (PFCN), and 12 electrons in 12 orbitals (**4a**-H) were used, while in the QD-NEVPT2 calculations an active space of 10 electrons in 10 orbitals was employed for both systems.

**Method**	**PFCN**	**4a-H**
ODM2/MRCI	3.77 (0.4611)	3.65 (0.8494)
QD-NEVPT2/def2-TZVP	3.49 (0.2606)	3.20 (0.6724)
TD-DFT wB97x-D/def2-TZVP	3.26 (0.6896)	3.37 (0.6918)
TDA TD-DFT wB97x-D/def2-SVP	3.32 (0.4609)	3.43 (0.5180)
TDA TD-DFT CAMB3LYP-BJ/def2-SVP	3.59 (0.6963)	3.58 (0.6809)
Exp.	—	3.18

Table S7: **Absorption properties of S and M forms of PFCN calculated at the TDA TD-DFT CAM-B3LYP-BJ/def2-SVP level.** They are calculated on the example of the $\mathbf{S}_M$ and $\mathbf{M}_M$ structures, optimized with the B3LYP-BJ/def2SVP method. E_{ex} is excitation energy of the $S_0 \rightarrow S_i$ transition (eV), f is the corresponding oscillator strength, and El. config. is the leading electronic configuration change.

**Transition**	E_{ex}	f	**El. config.**
**S**			
$S_0 \rightarrow S_1$	3.59	0.6963	H → L (93%)
$S_0 \rightarrow S_2$	3.72	0.0454	H-1 → L (89%)
**M**			
$S_0 \rightarrow S_1$	3.59	0.7597	H → L (94%)
$S_0 \rightarrow S_2$	3.76	0.0169	H-1 → L (90%)

Table S8: **Molecular orbitals participating in lowest-energy electronic transitions in PFCN,** calculated at the TDA TD-DFT CAM-B3LYP-BJ/def2-SVP level of theory for **S** and **M**, on the example of the $\mathbf{S}_M$ and $\mathbf{M}_M$ form, respectively

HOMO-1 HOMO LUMO

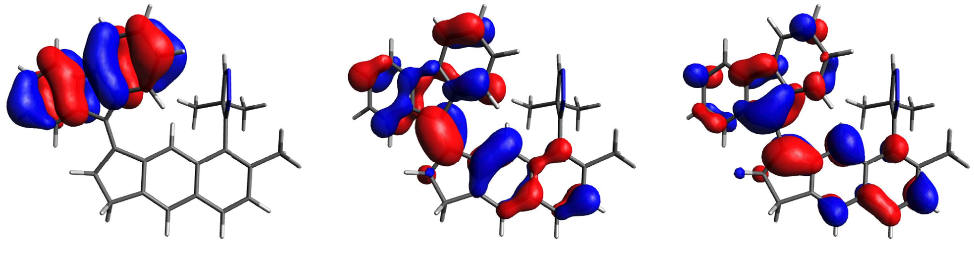

S

HOMO-1 HOMO LUMO

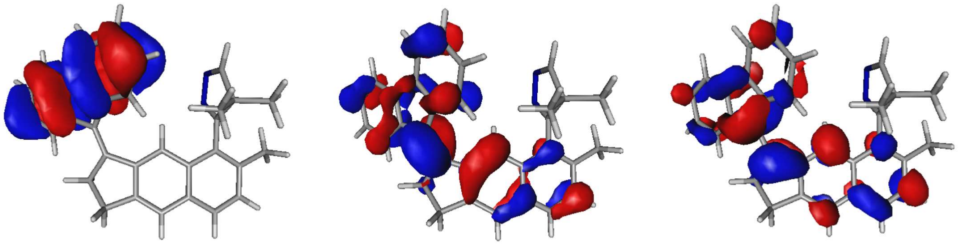

M

Table S9: **Absorption properties of S form of PFCN calculated at MRSF TD-DFT (CAM-B3LYP/cc-pVDZ) level of theory,** on the example of the $\mathbf{S}_M$ structure. E_{ex} is excitation energy $S_0 \rightarrow S_i$, λ is excitation energy expressed as wavelength, f is oscillator strength of transition.

**Transition**	E_{ex} **/ eV**	λ **/ nm**	f	**El. config.**
$S_0 \rightarrow S_1$	3.43	361	0.9988	H → L (91%)
$S_0 \rightarrow S_2$	3.51	353	0.0646	H-1 → L (89%)

Table S10: **Molecular orbitals participating in the electronic transitions of S form of PFCN calculated at the MRSF TD-DFT (CAM-B3LYP/cc-pVDZ) level of theory,** plotted on the example of the $\mathbf{S}_M$ structure.

HOMO-1 HOMO LUMO

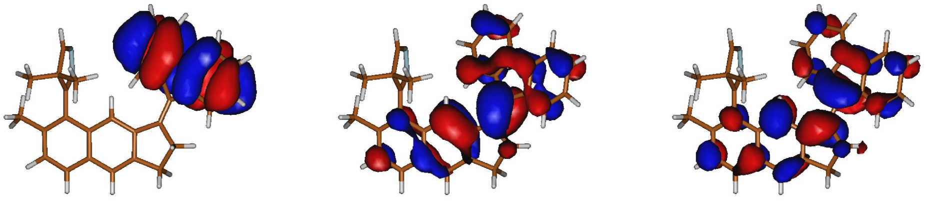

Table S11: **Adiabatic energies and characteristic structural parameters defined in Fig. S2 for optimized S_1 minimum and at S_1/S_0 MECI structures of PFCN.** Energies of the excited-state structures (E_{rel}, in eV) are given relative to the ground-state **S**-form energy at the corresponding level of theory (MRSF CAM-B3LYP/cc-pVDZ or ODM2/MRCI), while in parentheses the vertical S_1/S_0 energy gaps are reported. The dihedral angle values are given in degrees.

	**MRSF**	**ODM2/MRCI**		
	S_1	S_1	$MECI_{in}$	$MECI_{out}$
E_{rel}	2.481 (0.616)	2.775 (0.865)	3.041 (0.001)	3.120 (0.001)
θ	-102	-103	-25	-100
φ	-94	-77	-54	-136
α	-1	-15	-38	38

Table S12: **Absorption properties of the S and M forms of PFCN calculated with ODM2/MRCI in solution included at the COSMO level.** The UV-Vis absorption properties are calculated on the example of the $\mathbf{S}_M$ and $\mathbf{M}_M$ structures, optimized with ODM2/MRCI in vacuum. E_{ex} is excitation energy of the $S_0 \rightarrow S_i$ transition (eV), f is the corresponding oscillator strength, and Transition Character describes localization of the transition, where: Mot – whole motor molecule, Rot – the rotor part.

Solvent	E_{ex}	f	Transition character
**S:** $S_0 \rightarrow S_1$			
—	3.770	0.4641	Mot → Mot
Hexane	3.765	0.4662	Mot → Mot
DCM	3.758	0.4703	Mot → Mot
**S:** $S_0 \rightarrow S_2$			
—	4.169	0.0052	Rot → Mot
Hexane	4.195	0.0057	Rot → Mot
DCM	4.238	0.0068	Rot → Mot
**M:** $S_0 \rightarrow S_1$			
—	3.765	0.4788	Mot → Mot
Hexane	3.758	0.4801	Mot → Mot
DCM	3.744	0.4831	Mot → Mot
**M:** $S_0 \rightarrow S_2$			
—	4.204	0.0050	Rot → Mot
Hexane	4.220	0.0056	Rot → Mot
DCM	4.245	0.0068	Rot → Mot

Table S13: **Absorption properties of the S and M forms of PFCN calculated with TD-DFT wB97x-D/def2-TZVP in solution included at the COSMO level.** The UV-Vis absorption properties are calculated on the example of the $\mathbf{S}_M$ and $\mathbf{M}_M$ structures, optimized with ODM2/MRCI in vacuum. E_{ex} is excitation energy of the $S_0 \rightarrow S_i$ transition (eV), f is the corresponding oscillator strength, and Transition Character describes localization of the transition, where: Mot – whole motor molecule, Rot – the rotor part.

**Solvent**	E_{ex}	f	**Transition character**
**S:** $S_0 \rightarrow S_1$			
—	3.264	0.4499	Mot → Mot
Hexane	3.220	0.5505	Mot → Mot
DCM	3.226	0.5565	Mot → Mot
**S:** $S_0 \rightarrow S_2$			
—	3.596	0.0085	Rot → Mot
Hexane	3.628	0.0101	Rot → Mot
DCM	3.691	0.0125	Rot → Mot
**M:** $S_0 \rightarrow S_1$			
—	3.245	0.4700	Mot → Mot
Hexane	3.196	0.5714	Mot → Mot
DCM	3.199	0.5760	Mot → Mot
**M:** $S_0 \rightarrow S_2$			
—	3.619	0.0061	Rot → Mot
Hexane	3.642	0.0078	Rot → Mot
DCM	3.692	0.0094	Rot → Mot
